# SIFA: A two-stage adaptive ensemble framework for solar irradiance forecasting using a wrapper-based feature selection and chaotic manta ray optimization

**DOI:** 10.1038/s41598-026-53183-2

**Published:** 2026-06-10

**Authors:** Mohamed Abdel-Basset, Reda Mohamed, Ibrahim Alrashdi, Mahmoud Mahdi

**Affiliations:** 1 Faculty of Computers and Informatics, Zagazig University, Zagazig, 44519 Sharqiyah Egypt; 2 Department of Computer Science, College of Computer and Information Sciences, Jouf University, Sakaka, 2014 Saudi Arabia

**Keywords:** Machine Learning, Chaotic optimization, Solar irradiance, Forecasting, Feature selection, Hyperparameter tuning, Energy science and technology, Engineering, Mathematics and computing

## Abstract

Accurate solar irradiance forecasting is increasingly crucial for managing solar energy systems effectively, as their power output is directly dependent on solar irradiance (SI). Several models in the literature have been presented for SI forecasting; however, they still face at least one of these limitations: difficulties in modelling nonlinear data, demanding high computational resources, and often struggling to identify the best feature subsets for higher accuracy. To address these challenges, this study proposes a new multi-stage forecasting approach, termed SIFA, for accurate SI prediction, aiming to enhance the stability and efficiency of PV power plants. This approach comprises two main stages. The first stage employs a hybrid feature selection strategy combining random forest (RF) and sequential forward selection (SFS) to identify the most informative features. Specifically, SFS explores candidate feature subsets, while RF evaluates various subsets to select the most effective one. To further improve the RF performance, the number of estimators is tuned using an enhanced manta ray foraging optimizer, called IWMRFO, which employs chaotic maps instead of random generators to better balance exploration and exploitation, thereby avoiding local optima and accelerating convergence. The second stage combines three effective ML models—Huber Regressor (HR), Extra Trees (ET), and Extreme Gradient Boosting (XGB)—using a weight vector that is used to control the contribution of each base model in the hybrid ensemble approach (SIFA). This vector is optimized by the proposed IWMRFO, resulting in an adaptive ensemble that enhances predictive accuracy while maintaining stable generalization capability. This approach is tested on three popular datasets: the San Diego dataset, the Islamabad dataset, and the NASA SI dataset. Its performance is compared with several other models using several performance metrics, including RMSE, MAE, MAPE, MSE, and R². The numerical results demonstrate that SIFA achieved lower average forecasting errors than the competing models across the three evaluated datasets under repeated experiments, indicating that it is a strong alternative for predicting SI with higher accuracy.

## Introduction

 Conventional fossil fuels are the primary source of energy worldwide. However, they face the threat of depletion due to rapid consumption and the lengthy and costly process required to produce new fossil fuels^1^. Furthermore, it is the primary source of greenhouse gas emissions, such as carbon dioxide (CO₂), nitrous oxide (N₂O), and others. These emissions lead to climate changes, which threaten the environment and people’s lives^2^. Hence, finding new energy sources has become essential to combat climate change driven by traditional fossil fuels and to fulfill increasing energy needs as fossil fuel reserves dwindle^3,4^. Therefore, the countries have recently shifted toward renewable energy sources, such as wind, solar, and hydropower, which are derived from natural processes and have become essential alternatives due to their continuous generation, low costs, and significantly lower environmental impact. Among renewable energy sources, solar energy has attracted significant attention because of its reliability, low operating costs, and dependence on solar irradiance (SI)^4^. Accurate SI forecasting is increasingly crucial for managing solar energy systems effectively, since their power output depends on solar irradiance. The SI forecasting process is categorized into four groups based on forecast horizons: very short-term (critical for system monitoring), short-term (important for decision-making such as balancing demand and supply, committing units, and more), medium-term (used for planning maintenance schedules for power plants), and long-term (essential for site selection, solar plant installation, network operations, and related tasks)^5^.

Accurately predicting SI, especially in the short-term horizon, is essential for the stable and efficient operation of PV power plants. Therefore, in the literature, several SI forecasting models, divided into statistical models and machine learning (ML) and deep learning (DL) models, have been presented recently for handling this problem^6^. This study focuses on ML and DL models, as they are the most effective at capturing nonlinear dynamic relationships in SI data. Therefore, this section reviews recent studies on SI forecasting to highlight their strengths and weaknesses while identifying gaps that justify our study. Ahmed et al.^6^ introduced an ensemble SI forecasting model called PBNN, which combines three ML models—RF, XGB, and Categorical Boosting (CatBoost)—with a feedforward neural network (FFNN). The ML models serve as base learners trained on datasets, and their outputs are weighted by the FFNN to generate final predictions. Mutual information was used to select the best features, boosting the PBNN’s performance. The model was evaluated with two datasets—Islamabad and San Diego—and compared against several other models using various performance metrics, demonstrating its effectiveness. Results showed it outperformed all rivals, with improvements of approximately 47% and 74% for the respective datasets. However, it still risks getting stuck in local optima due to dependence on gradient-based optimizers for determining the optimal weights of the ML models.

This study^7^ presents a reliable DL framework for analyzing multi-scale variations in solar radiation. It combines feature engineering with transformation matrices, uses CNNs for local feature detection, and LSTMs for global uncertainty modeling. Trained on Tokyo meteorological data, the model shows high accuracy with an R² of 0.97 on unseen data. Zhu et al.^8^ presented a hybrid approach for predicting direct normal irradiance. This approach combined decomposition, clustering, the battle royale optimizer, and the CatBoost algorithm. Data from China’s Jiangsu province across all seasons were analyzed. Features were selected, decomposed via wavelet analysis, and grouped using sample entropy. The data were input into an optimized CatBoost model fine-tuned by the battle royale optimizer. This hybrid model could achieve outstanding results compared to several rival models. Díaz-Bedoya et al.^9^ explored SI forecasting with DL and ensemble models, using LSTM, RF, and ETs with meteorological data to improve accuracy. Zhang^10^ develops a hybrid forecasting model that combines sample entropy (SE) clustering, the CEEMDAN decomposition technique, support vector regression (SVR), and the grey wolf optimizer (GWO). Based on data from Jiangsu, China, the CEEMDAN-SE-GWO-SVR model outperforms the multi-layer perceptron (MLP) and the LSTM.

Naveed et al.^11^ introduced three innovative models for SI prediction: SEFMNN, Extreme Learning Machine (ELM), and XGB-Squared Error (XGB-SE). These models forecast shortwave SI in three Chinese regions—Guangdong, Shandong, and Zhejiang—and one in Saudi Arabia, Najran. SEFMNN integrates ANN, RF, and SVR to boost accuracy, while XGB-SE incorporates a specialized loss function for handling extreme data. All models aim to reduce overfitting and enhance efficiency and precision. Both XGB-SE and SEFMNN outperform ELM, significantly advancing SI forecasting for PV system planning. In^12^, various ML models, including linear regression, RF, decision trees, SVR, and gradient boosting, were tested for SI prediction. The simulation results demonstrate that RF is more effective, achieving an RMSE of 0.64 and surpassing linear regression by 19.47%. Mariappan^13^ introduced a hybrid DL model, CNN-SLSTM, to forecast daily global SI using measurement data and weather information. The recursive feature elimination technique based on RF was used to select the best subset of 14 key features. The hyperparameters of CNN-SLSTM were optimized using the slime mold algorithm. Through ten-fold cross-validation, the model was compared to several DL models, such as Gated Recurrent Unit (GRU), LSTM, CNN-LSTM, and various ML regressors like RF, SVR, and Decision Tree. The results indicate that CNN-SLSTM outperformed the other models.

Raju et al.^14^ introduced a hybrid DL model that combines LSTM networks with Chaotic Particle Swarm Optimization (CPSO). The DL model employs LSTM to identify complex temporal patterns in short-wave SI data. CPSO fine-tunes LSTM hyperparameters such as neurons, learning rate, batch size, dropout, and activation function to minimize prediction errors. The model’s performance is evaluated using metrics such as MAE, MAPE, RMSE, and the coefficient of determination. The best results are achieved for 60-minute-ahead forecasts during the rainy season. Li et al.^15^ introduced a Shadow-Attention Graph Neural Network (SAGNN) to predict SI for urban buildings. Alorf^16^ proposed an N-hours-ahead SI forecasting method that integrates variational mode decomposition (VMD) with an enhanced temporal fusion transformer (TFT). The approach employs VMD to decompose data into intrinsic mode functions (IMFs) and enhances TFT by incorporating a screening network and a GRU encoder–decoder. It can forecast at 1-hour intervals and other horizons. The experimental results demonstrate that this method outperforms several rival models.

Prajesh^17^ introduced the Light Gradient Boosting Machine (LightGBM), an ensemble learning method that reduces computation time and enhances forecasting accuracy. It employs a two-step process: using mutual information for feature selection, followed by an autoencoder for feature extraction to prevent overfitting. Additionally, the hyperparameters of LightGBM are tuned using the Honey Badger Algorithm to improve its performance on the SI forecasting problem. Testing on two datasets from the National Renewable Energy Laboratory demonstrates its advantages over benchmark models. In^18^, a novel GRU-TCN model that integrates a temporal convolutional network (TCN) and a GRU was proposed. It captures temporal features from SI data using the GRU and spatial features with the TCN. Both univariate and multivariate GRU-TCN models are used for ultra-short-term SI forecasting and are compared to several models, such as TCN, LSTM, and GRU. The empirical results show that the univariate model based on historical SI data is the best. Several other SI approaches have been recently presented for the SI forecasting, some of them are multivariate ML models^19^, convolution neural network-LSTM (CNN-LSTM)^20^, hybrid DL model^21^, EEMD–Transformer–GRU model^22^, SVR and Gaussian Process (GP) techniques^23^, Hybrid DL CNN-LSTM^24^, U-Shaped LSTM-Attention-Free Transformer (AFT)^25^, hybrid quantum neural network^26^, stacked ensemble learning-based correction model^27^, Quantum Neural Network^28^, a hybrid transformer-based framework^29^, ANN optimized by the Coati optimization algorithm^30^, and others^31–41^.

Although several studies have explored SI forecasting, they still struggle with modeling nonlinear data, require substantial computational costs, and often fail to select the optimal feature subsets to improve accuracy. Therefore, this study introduces a new multi-stage forecasting model called SIFA to accurately predict the SI, enhancing the stability and efficiency of PV power plants. The idea behind this model is based on employing a modern metaheuristic algorithm called the manta ray foraging optimizer (MRFO) to determine each base model’s contribution, which could lead to better predictive accuracy. We considered the metaheuristic algorithm due to its recent successful application in various ML tasks, achieving excellent results in a shorter time. Some of these tasks include student academic performance prediction^42^, anomaly detection in IoT networks^43^, ELM training tasks for medical datasets^44^, Power Quality Event Classification^45^, accurate diagnosis of Sjögren’s syndrome^46^, and feature selection tasks^47–50^. It is also worth noting that we chose the metaheuristic approach over the ANN employed in^6^, as the latter is more prone to falling into local optima and requires higher computational costs, as confirmed in our later experiments. Although several metaheuristic algorithms have been proposed, such as the Secant Optimization Algorithm^51^, Schrödinger optimizer^52^, and others^53^, we use MRFO in this study because it demonstrates stable and effective performance when handling various optimization problems in the literature.

The proposed SIFA approach consists of two stages: the feature selection stage using the RF-based sequential forward selection (SFS) method and optimized weight-based hybrid ML models. In the first stage, we use the SFS method to choose the optimal feature subset for improving SI predictions. This approach is integrated with the RF technique to evaluate various selected subsets, ultimately identifying the most effective one that leads to more accurate predictions. The number of estimators in the RF model as a hyperparameter affects the selection process. Tuning this hyperparameter is essential to improve the selected feature subset. Therefore, in this study, we present an enhanced version of the manta ray foraging optimizer (MRFO) that uses chaotic maps instead of traditional random generators to balance exploration and exploitation during optimization, called IWMRFO. This helps prevent stagnation in local optima and enhances convergence speed. This improved variant is used to tune the number of estimators in the RF model to achieve the optimal feature subset. The second stage involves using weight-based ML models to predict the SI based on the best feature subset. This stage combines three effective ML models—HR, ET, and XGB—based on a weight vector. This vector assigns a weight to each of the three models to determine their contribution to prediction accuracy. Estimating this weight vector is considered an optimization problem that must be solved accurately to determine each model’s contribution to improved predictive accuracy. Therefore, we employ the proposed IWMRFO to handle this problem. It is worth mentioning that ET, HR, and XGB are selected due to their effectiveness in modeling nonlinear features in the SI data. The proposed SIFA approach is evaluated using three popular datasets— the San Diego dataset, the Islamabad dataset, and the NASA SI dataset—and compared to several competing models based on various performance metrics: RMSE, MAE, MAPE, MSE, and R². SIFA, according to the experimental results, could significantly outperform all models across all performance metrics on the three datasets. The main contributions of this study are listed below:

a) Introducing A two-stage adaptive ensemble framework (SIFA) for accurate SI forecasting.

b) The first stage employs a wrapper-based feature selection method based on the RF-based SFS method to select the most informative features.

c) The second stage integrates three effective ML models HR, ET, and XGB using a weight vector to control the contribution of each base model in SIFA.

d) Tuning the number of estimators in the RF-based SFS method and the weight vector using an improved version of MRFO that employs chaotic maps instead of traditional random generators to balance exploration and exploitation during optimization.

e) Using three popular datasets San Diego, Islamabad, and NASA SI to evaluate the proposed approach, along with comparing it to several competing models based on various performance metrics: RMSE, MAE, MAPE, MSE, and R².

f) SIFA, based on the experimental results, was able to show lower error variability than all compared models on the three datasets, with improvements ranging from 10% to 95%.

This study is organized as follows: Sect.  2 introduces the enhanced MRFO, Sect.  3 explains the ET, XGB, and HR models, Sect.  4 outlines the proposed model, Sect.  5 details the experimental settings, Sect.  6 presents the results and discussion, and Sect.  7 presents conclusions and future research directions.

### Enhanced manta ray foraging optimizer: WMQIMRFO

Recently, a new optimization algorithm called the MRFO, inspired by manta rays’ foraging behaviors chain, cyclone, and somersault—was developed for engineering optimization challenges^54^. In MRFO, the chain foraging behavior involves moving all individuals, except the first, towards both the individual in front of them and the best solution found so far. Meanwhile, the first individual is moved solely towards the best-so-far solution. This behavior is mathematically defined as follows:

1 $$\:\vec{x}_{i}^{{t + 1}} = \left\{ {\begin{array}{*{20}c} {\vec{x}_{i}^{t} + \vec{r} \cdot \:\left( {\vec{x}^{{\mathrm{*}}} - \vec{x}_{i}^{t} } \right) + \alpha \cdot \:\left( {\vec{x}^{{\mathrm{*}}} - \vec{x}_{i}^{t} } \right),\:\:\:\:\:\:\:\:\:\:\:\:\:\:\:\:\:if\:i = 1} \\ {\:\vec{x}_{i}^{t} + \vec{r} \cdot \:\left( {\vec{x}_{{i - 1}}^{t} - \vec{x}_{i}^{t} } \right) + \alpha \: \cdot \left( {\vec{x}^{{\mathrm{*}}} - \vec{x}_{i}^{t} } \right),\:\:otherwise} \\ \end{array} } \right.$$

2 $$\:\alpha \: = 2 \cdot \:r\: \cdot \sqrt {\left| {{\mathrm{log}}r} \right|}$$

where $\:\overrightarrow{r}$ is a random vector in $\:[0,\:1]$, $\:{\overrightarrow{x}}^{*}$ stands for the best solution found so far, $\:{\overrightarrow{x}}_{i}^{t+1}$ is the new solution, and $\:{\overrightarrow{x}}_{i}^{t}$ is the current solution. The MRFO’s cyclone foraging mechanism consists of two parts. The first part emphasizes exploring areas near $\:{\overrightarrow{x}}^{*}$ to accelerate convergence and is expressed as follows:

3 $$\:\vec{x}_{i}^{{t + 1}} = \left\{ {\begin{array}{*{20}c} {\vec{x}^{{\mathrm{*}}} + \vec{r} \cdot \:\left( {\vec{x}^{{\mathrm{*}}} - \vec{x}_{i}^{t} } \right) + \beta \: \cdot \:\left( {\vec{x}^{{\mathrm{*}}} - \vec{x}_{i}^{t} } \right),\:\:\:\:\:\:\:\:\:\:\:\:\:\:\:\:\:if\:i = 1} \\ {\:\vec{x}^{{\mathrm{*}}} + \vec{r} \cdot \:\left( {\vec{x}_{{i - 1}}^{t} - \vec{x}_{i}^{t} } \right) + \beta \: \cdot \:\left( {\vec{x}^{{\mathrm{*}}} - \vec{x}_{i}^{t} } \right),\:\:otherwise} \\ \end{array} } \right.$$

4 $$\:\beta \: = 2 \cdot \:e^{{r_{1} \frac{{T_{{max}} - t + 1}}{{T_{{max}} }}}} \cdot {\mathrm{sin}}\left( {2\pi \:r_{1} } \right)$$

where $\:{r}_{1}$ represents a number randomly chosen in $\:[0,\:1]$, $\:t$ stands for the current iteration, and $\:{T}_{max}$ stands for the maximum iteration. On the other hand, the second part emphasizes thoroughly exploring the search space to avoid getting stuck in local optima and improve the final results. This part is described as follows:

5 $$\:\vec{x}_{i}^{{t + 1}} = \left\{ {\begin{array}{*{20}c} {\vec{x}_{a}^{t} + \vec{r} \cdot \left( {\vec{x}_{a}^{t} - \vec{x}_{i}^{t} } \right) + \beta \: \cdot \:\left( {\vec{x}_{a}^{t} - \vec{x}_{i}^{t} } \right),\:\:\:\:\:\:\:\:\:\:\:\:\:\:\:\:\:if\:i = 1} \\ {\:\vec{x}_{a}^{t} + \vec{r} \cdot \:\left( {\vec{x}_{{i - 1}}^{t} - \vec{x}_{i}^{t} } \right) + \beta \: \cdot \:\left( {\vec{x}_{a}^{t} - \vec{x}_{i}^{t} } \right),\:\:otherwise} \\ \end{array} } \right.$$

6 $$\:{\overrightarrow{x}}_{a}^{t}=\overrightarrow{L}+\overrightarrow{r}\cdot\:\left(\overrightarrow{U}-\overrightarrow{L}\right)$$

where$\:\:\overrightarrow{L}$ stands for the lower bound, and $\:\overrightarrow{U}\:$denotes the upper bound.

However, the second part was enhanced in^55^ by integrating it with the Morlet wavelet mutation (MWM) strategy to further strengthen the exploration operators and avoid stagnation in local optima. This strategy is used to update the current solutions based on a mutation probability ($\:{p}_{m}$). When this probability is smaller than a number randomly chosen in $\:[0,\:1]$, the MWM strategy is implemented; otherwise, the traditional chain foraging equation defined in (5) is applied. The mathematical model of this strategy is as follows:

7 $$\vec{x}_{i}^{{t + 1}} = \left\{ {\begin{array}{*{20}c} {\vec{x}_{a}^{t} + \vec{r} \cdot \left( {\vec{x}_{a}^{t} - \vec{x}_{i}^{t} } \right) + \beta \cdot \left( {\vec{x}_{a}^{t} - \vec{x}_{i}^{t} } \right) + \sigma \cdot \left( {\vec{U} - \vec{x}_{i}^{t} } \right),~~~~~~~~~~~~~~~~~if~i = 1} \\ {\vec{x}_{a}^{t} + \vec{r} \cdot \left( {\vec{x}_{{i - 1}}^{t} - \vec{x}_{i}^{t} } \right) + \beta \cdot \left( {\vec{x}_{a}^{t} - \vec{x}_{i}^{t} } \right) + \sigma \cdot \left( {\vec{x}_{i}^{t} - \vec{L}} \right),~~otherwise} \\ \end{array} } \right.$$

8 $$\sigma = 1/\sqrt a \psi \left( {\varphi /a} \right)$$

9 $$a = s \cdot \left( {\frac{1}{s}} \right)^{{\left( {1 - \frac{t}{{T_{{max}} }}} \right)}}$$

where $\:s$ is a constant value,$\:\:\varphi\:$ is a number selected at random in the interval of $\:-2.5a$ and $\:2.5a$, and $\:\psi\:\left(\varphi\:/a\right)$ represents the Morlet wavelet function:

10 $$\:\psi\:\left(x\right)={e}^{-{x}^{2}/2}\mathrm{cos}\left(5x\right)$$

According to^54^, the preference for both parts during the optimization process depends on the current iteration, with the first part applied initially and the second part introduced as the optimization process advances. This implies that their preference is linearly weighted throughout the optimization, possibly leading to slow convergence in the early stages and stagnation in local optima later. To address this, the authors in^55^ adopted a nonlinear approach to enhance exploration and exploitation during different optimization phases. They employ sine, cosine, tangent, and logarithmic functions, defined as follows:

11 $$\:{C}_{f}=\mathrm{sin}\left(\frac{\pi\:}{2}\cdot\:\frac{t}{{T}_{max}}\right)$$

12 $$\:{C}_{f}=1-\mathrm{cos}\left(\frac{\pi\:}{2}\cdot\:\frac{t}{{T}_{max}}\right)$$

13 $$\:{C}_{f}=\mathrm{tan}\left(\frac{\pi\:}{4}\cdot\:\frac{t}{{T}_{max}}\right)$$

14 $$\:{C}_{f}=\mathrm{ln}\left(1+\left(e-1\right)\frac{t}{{T}_{max}}\right)$$

Finally, the tradeoff between both parts, according to one of the nonlinear approaches mentioned above, is defined as follows:

15 $$\:{\overrightarrow{x}}_{i}^{t+1}=\left\{\begin{array}{c}\left\{\begin{array}{c}\left(5\right),\:\:\:r<{p}_{m}\\\:\left(7\right),\:\:\:r\ge\:{p}_{m}\end{array}\right.\:\:\:\:\:\:\:\:\:\:\:\:\:\:\:\:\:\:\:\:\:\:\:\:\:if\:{r}_{1}>{C}_{f}\\\:\left(3\right),\:\:\:\:\:\:\:\:\:\:\:\:\:\:\:\:\:\:\:\:\:\:\:\:otherwise\end{array}\right.\:$$

where $\:r$ and $\:{r}_{1}$ are numbers randomly chosen in $\:[0,\:1]$. Finally, the somersault foraging mechanism consistently updates the individual based on the best solution identified so far, enhancing exploitation and speeding up convergence to the global optimum. This mechanism is defined by:

16 $$\:\vec{x}_{i}^{{t + 1}} = \:\vec{x}_{i}^{t} + S \cdot \:\left( {r_{2} \cdot \:\vec{x}^{{\mathrm{*}}} - r_{3} \cdot \:\vec{x}_{i}^{t} } \right)$$

where $\:S$ represents the somersault factor and is advised to be configured at 2, and $\:{r}_{2}$ and $\:{r}_{3}$ represent numbers randomly chosen in $\:[0,\:1]$. To further enhance the exploitation operator of the MRFO algorithm, a quadratic interpolation (QI) strategy is integrated into the updated population, as described in^55^, to accelerate convergence and improve accuracy. Finally, integrating the QI, MWM, and nonlinearity mechanisms with the standard MRFO could produce a more effective variant, called WMQIMRFO (referred to as WMRFO for short), featuring stronger exploration and exploitation operators in the optimization process to improve convergence speed and prevent stagnation in local optima.

### Machine learning models: Overview

This section reviews the core ML models employed in developing the proposed model, including HR, XGB, and ET regression.

### Huber regression model

In 1964, Peter J. Huber introduced the Huber Regressor (HR) to address the important issue of sensitivity in linear regression. HR combines the advantages of linear and robust regression for reducing the influence of outliers on the model’s accuracy. It uses the Huber loss function, which acts quadratically for small residuals—similar to ordinary least squares—and linearly for large residuals, like robust regression, ensuring efficiency while reducing sensitivity to outliers^56^. The Huber loss function is defined by

17 $$\:H\left({r}_{i}\right)=\left\{\begin{array}{c}\frac{1}{2}{r}_{i}^{2}\:\:\:\:\:\:\:\:\:\:\:\:\:\:\:\:\:\:\:\:\:\:\:\:if\:\left|{r}_{i}\right|\le\:\eta\:\\\:\eta\:\left(\:\left|{r}_{i}\right|-\frac{1}{2}\:\eta\:\right)\:\:\:Otherwise\end{array}\right.$$

where $\:{r}_{i}$ represents the difference between the predicted and estimated values for the ith sample, and $\:\eta\:$ is a threshold parameter that defines the point at which the loss function switches from quadratic to linear. The HR model involves hyperparameters like $\:\eta\:$ in (17), max_iter for weight updates, and alpha for regularization to prevent overfitting. Accurate estimation of these hyperparameters is crucial for improving HR’s prediction for SI.

### Extra trees regression

**Fig. 1:**
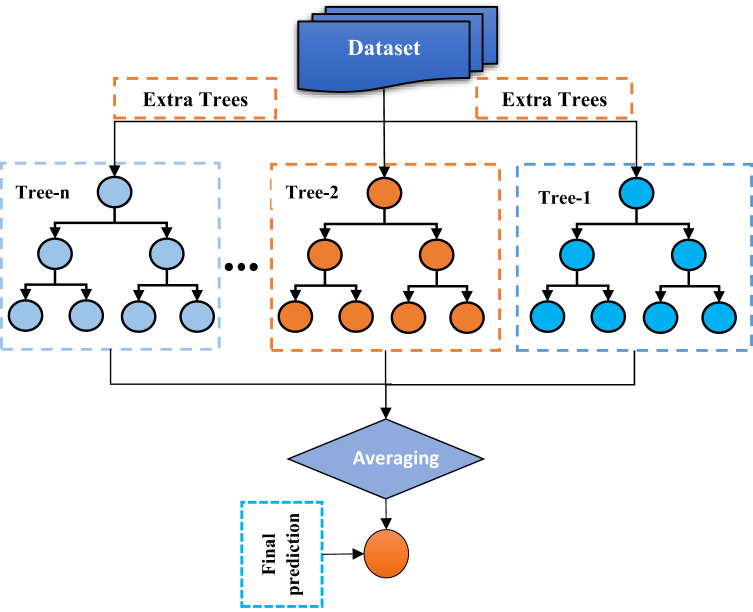
ET regression’s architecture.

The ET regression model is an ensemble learning method similar to RF, applicable for both classification and regression^57^. As shown in Fig. 1, ET consists of several decision trees (DTs). Unlike RF, where each tree is trained on a bootstrap sample, ET builds all trees using the entire training dataset. ET adds stochasticity by randomly choosing split thresholds for each feature, which improves generalization and reduces overfitting. The final prediction results from averaging the outputs of all DTs, as detailed in the following equation^57^:

18 $$\:{F}_{p}=\frac{1}{n}\sum\:_{i=1}^{n}{T}_{i}\left(\overrightarrow{x}\right)$$

where $\:{F}_{p}$ denotes the final forecast for the input sample ($\:\overrightarrow{x}$), $\:n$ is the number of decision trees in the ensemble, and $\:{T}_{i}$ represents the output of the $\:ith$ decision tree for $\:\overrightarrow{x}$.

### Extreme gradient boosting

Boosting algorithms combine weak classifiers to create a strong learner that improves data interpretation and increases prediction or classification accuracy^58^. There are several boosting algorithms found in the literature, such as Adaboost^59^, histogram-based gradient boosting (HGB), gradient boosting^60^, CatBoost^61^, and XGB. The XGB algorithm is an optimized and scalable gradient boosting method aimed at achieving high predictive accuracy and efficiency^62,63^. It constructs an ensemble of weak learners typically decision trees by adding each one sequentially, with each new tree focusing on correcting the residual errors from previous trees. The model utilizes a regularized loss function that combines a differentiable error metric with a regularization term, which helps limit the model’s complexity. This approach reduces the risk of overfitting and improves the model’s ability to generalize. XGB includes several algorithmic improvements, such as second-order gradient optimization, shrinkage (learning rate), and parallelized tree building. These enhancements increase its accuracy and speed. As a result, XGB has become a leading ensemble method for both classification and regression tasks, particularly in large-scale and high-dimensional datasets. The final model prediction is computed by summing the outputs of all trees, as defined in the following formula:

19 $$\:{F}_{p}^{k}=\sum\:_{i=1}^{n}{T}_{i}\left(\overrightarrow{{x}_{k}}\right)$$

where $\:{F}_{p}^{k}$ stands for the predicted value for the $\:kth$ sample, and $\:{T}_{i}$ represents the forecasting of the $\:ith$ tree for the $\:kth$ sample.

### Proposed model: SI forecasting approach (SIFA)

This section explains the proposed methodology, which combines three effective ML models whose weights are optimized by the enhanced WMRFO. These optimized weights determine each model’s contribution to the final predictions, enabling the selection of a combination with strong generalization ability and high predictive accuracy. Additionally, in the preprocessing step, we use the RF-based sequential forward selection (SFS) technique to choose the optimal subset of features, thereby improving the model’s performance. The number of estimators in the RF—a hyperparameter affecting the model’s performance—is optimized using the proposed IWMRFO. Overall, the proposed model involves feature selection with RF-based SFS, IWMRFO enhanced with chaotic maps, and a weighted hybrid ML. These steps are explained in detail in the following subsections.

### RF-based SFS technique: The feature selection step

The feature selection step is crucial for several ML tasks because of its role in improving classification accuracy and reducing computational costs. This step is performed using various techniques divided into three classes: wrapper-based, filter-based, and embedded-based. Filter-based techniques use statistical metrics to assess the correlation between each independent feature and the target variable. These methods rely on measures like correlation coefficients, mutual information, chi-square statistics, or ANOVA F-values to gauge the strength of the association; therefore, they are known as model-independent methods. Features that exhibit little or no correlation with the target are deemed irrelevant and are removed from the dataset. The wrapper-based method is dependent on the model, as it utilizes an ML model to evaluate the quality of the selected feature subset. The RF-based SFS method falls into this category, starting by evaluating each feature individually with RF and storing the one with the lowest MSE in a feature subset array. In the next iteration, the remaining features are tested one by one in combination with those already in the array, and any feature that improves performance is added to the array. This iterative process repeats until the number of required features is selected. Finally, the resulting subset comprises the most informative, non-redundant features identified by the RF model. However, the RF model has some hyperparameters that influence the quality of the selected features; the most important one is the number of estimators (decision trees). This hyperparameter needs to be estimated accurately to help select the best feature subset with the aim of improving predictive accuracy and reducing computational costs. Therefore, in this study, we use the proposed IWMRFO discussed in the next section to determine the optimal value for this parameter. Finally, the pseudocode for the RF-based SFS technique is provided in Algorithm 1. This algorithm aims to achieve high effectiveness in reducing dimensionality, eliminating redundancy, and enhancing model efficiency without compromising predictive accuracy.

**Algorithm 1:**
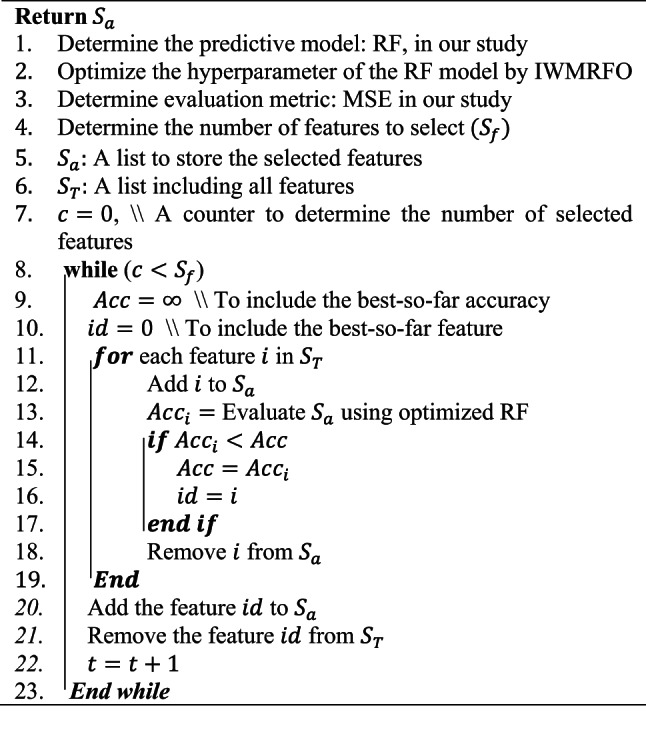
Pseudocode of RF-based SFS.

### Proposed chaotic-based WMRFO: IWMRFO

Chaotic maps can replace the WMRFO algorithm’s traditional pseudorandom numbers by generating a sequence of values using a deterministic recursive equation that begins with a randomly initialized seed value, improving the algorithm’s exploration ability and convergence stability. Chaotic sequences are deterministically random, exhibiting characteristics such as ergodicity and sensitivity to initial conditions, which help diversify the search process, thoroughly explore the search space, and reduce the risk of premature convergence to local optima. Additionally, the deterministic nature of chaos helps maintain stability during optimization. By substituting random distributions with chaotic sequences, WMRFO can better balance exploration and exploitation, resulting in improved convergence accuracy and higher-quality solutions. This study employs ten one-dimensional maps, commonly used in various literature sources^24,25^, to generate chaotic sets. Given their diverse behaviors, we compare the effectiveness of the proposed MWRFO algorithm with several of these chaotic maps to identify which yields the most accurate results. The mathematical definitions for these 10 chaotic maps are summarized in Table 1^64,65^. These maps are designed to replace some random numbers—not all—in WMRFO equations to encourage diversity in the algorithm’s search behaviors and achieve higher-quality solutions. The first is the random number in (15), which controls the balance between the two parts of the cyclone foraging mechanism. When chaotic maps are used, this equation is redefined as follows:

20 $$\:{\overrightarrow{x}}_{i}^{t+1}=\left\{\begin{array}{c}\left\{\begin{array}{c}\left(5\right),\:\:\:r<{p}_{m}\\\:\left(21\right),\:\:\:r\ge\:{p}_{m}\end{array}\right.\:\:\:\:\:\:\:\:\:\:\:\:\:\:\:\:\:\:\:\:\:\:\:\:\:if\:{C}_{i}>{C}_{f}\\\:\left(3\right),\:\:\:\:\:\:\:\:\:\:\:\:\:\:\:\:\:\:\:\:\:\:\:\:otherwise\end{array}\right.\:$$

where $\:{C}_{i}$ denotes a number generated by one of the ten chaotic maps. This update adjusts the balance between the exploration operators in (5–6) and the exploitation operators in (3), helping to prevent local optima traps and accelerating convergence.

In addition, the random vector in (7) is also replaced by the chaotic maps to improve the exploration operator along the optimization process. However, this equation will strongly motivate exploration operators, which can increase population diversity but slow down convergence. To address this, this equation is modified to dynamically steer the search: it emphasizes exploitation near $\:{\overrightarrow{x}}^{*}$ when the chaotic value is below 0.5 and encourages exploration around $\:{\overrightarrow{x}}_{a}^{t}$ when it exceeds 0.5. As the chaotic value nears 0.5, the method gradually balances exploration and exploitation. (7) after modification is defined as follows:

21 $$\:\vec{x}_{i}^{{t + 1}} = \left\{ {\begin{array}{*{20}c} {\overrightarrow {{x_{{Ba}} }} + \overrightarrow {{C_{3} }} \cdot \:\left( {\vec{x}_{a}^{t} - \vec{x}_{i}^{t} } \right) + \beta \: \cdot \:\left( {\vec{x}_{a}^{t} - \vec{x}_{i}^{t} } \right) + \sigma \: \cdot \:\left( {\vec{U} - \vec{x}_{i}^{t} } \right),\:\:\:\:\:\:\:\:\:\:\:\:\:\:\:\:\:if\:i = 1} \\ {\:\overrightarrow {{x_{{Ba}} }} + \overrightarrow {{C_{3} }} \cdot \:\left( {\vec{x}_{{i - 1}}^{t} - \vec{x}_{i}^{t} } \right) + \beta \: \cdot \:\left( {\vec{x}_{a}^{t} - \vec{x}_{i}^{t} } \right) + \sigma \: \cdot \:\left( {\vec{x}_{i}^{t} - \vec{L}} \right),\:\:otherwise} \\ \end{array} } \right.$$

22 $$\:\overrightarrow{{x}_{Ba}}={C}_{2}\mathrm{*}{\overrightarrow{x}}^{\mathrm{*}}+\left(1-{C}_{2}\right)\mathrm{*}{\overrightarrow{x}}_{a}^{t}$$

where $\:\overrightarrow{{C}_{3}}$ denotes vector generated by one of the ten chaotic maps, and $\:{C}_{2}$ denotes a number generated by one of the ten chaotic maps. The pseudocode for the enhanced WMRFO (IWMRFO) is outlined in Algorithm 2. It starts by creating an $\:N\:\times\:\:D$ matrix, where $\:N$ is the population size and $\:D$ is the problem’s dimension. This matrix is initialized randomly within the problem’s lower and upper bounds. All solutions in the matrix are then evaluated individually, and their fitness scores are compared. The solution with the highest accuracy is selected as the best so far. The population is subsequently refined using IWMRFO’s update schemes and re-evaluated to improve $\:{\overrightarrow{x}}^{*}$, as detailed in Lines 6–28. This cycle repeats until the maximum number of function evaluations is reached, as indicated in Line 7. Upon completing the optimization process, the final best-so-far solution is returned.

**Algorithm 2:**
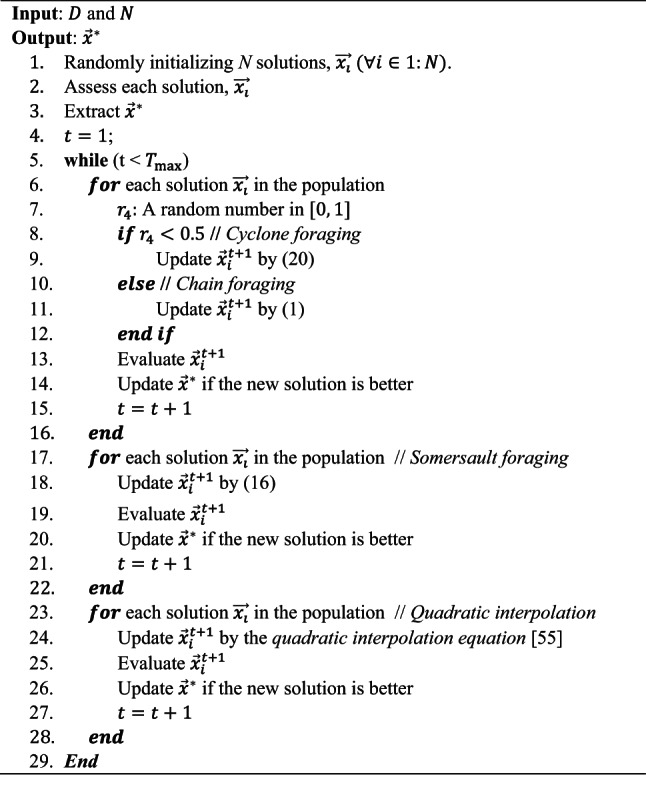
Pseudocode of IWMRFO.

**Table 1:** Description of the ten used chaotic maps.

Id	Name	Mathematical model	Parameters
C1	Chebyshev map	$\:x_{{i + 1}} = {\mathrm{cos}}\left[ {i \times \:acos\left( {x_{i} } \right)} \right] \cdot$	-
C2	Circle map	$\:x_{{i + 1}} = \left( {x_{i} + c_{1} - \frac{{c_{2} }}{{2\pi \:}}{\mathrm{sin}}\left( {2\pi \:x_{i} } \right)} \right)\:{\mathrm{mod}}\:1 \cdot$	$\:{c}_{1}=0.2$ $\:{c}_{2}=0.5$
C3	Gauss/mouse map	$$\:{x}_{i+1}=\left\{\begin{array}{c}1,\:\:\:\:\:\:\:\:\:\:{x}_{i}=0\\\:\left(\frac{1}{{x}_{i}}\right)\:mod\:1,\:\:otherwise\:\:\:\end{array}\right.\cdot\:$$	-
C4	Iterative map	$\:{x}_{i+1}=\mathrm{sin}\left[\frac{l\pi\:}{{x}_{i}}\right]\cdot\:$	$\:l=4$
C5	Logistic map	$\:{x}_{i+1}={c}_{3}{x}_{i}\left(1-{x}_{i}\right)\cdot\:$	$\:{c}_{3}=4$
C6	Piecewise map	$$\:{x}_{i+1}=\left\{\begin{array}{c}\frac{{x}_{i}}{l},\:\:\:\:\:\:\:\:\:\:\:\:\:\:\:\:\:\:\:0\le\:{x}_{i}<l\\\:\frac{{x}_{i}-l}{0.5-l},\:\:\:\:\:\:\:\:\:\:\:\:\:l\le\:{x}_{i}<0.5\\\:\frac{{1-l-x}_{i}}{0.5-l},\:\:\:\:\:0.5\le\:{x}_{i}<1-l\\\:\frac{1-{x}_{i}}{l},\:\:\:\:\:\:\:\:\:1-l\le\:{x}_{i}<1\end{array}\right.$$	$\:l=0.4$
C7	Sine map	$\:{x}_{i+1}=\mathrm{sin}\left[\pi\:{x}_{i}\right]\cdot\:$	**-**
C8	Singer map	$\:{x}_{i+1}=\mu\:\left(7.86{x}_{i}-23.31{x}_{i}^{2}+28.75{x}_{i}^{3}-13.302875{x}_{i}^{4}\right)\:\cdot\:$	$\:\mu\:=1.07$
C9	Singer map	$\:{x}_{i+1}=l{x}_{i}^{2}\mathrm{sin}\left(\pi\:{x}_{i}\right)\cdot\:$	$\:l=2.3$
C10	Sinusoidal map	$$\:{x}_{i+1}=\left\{\begin{array}{c}{x}_{i}/0.7\:\:\:\:{x}_{i}<0.7\\\:\mu\:\left({1-x}_{i}\right)\:\:\:{x}_{i}\ge\:0.7\end{array}\right.\:\cdot\:$$	$\:\mu\:=10/3$

### Optimized weight-based hybrid ML: SIFA

This study introduces a weight-based hybrid ML model (SIFA) that combines three robust algorithms: ET, HR, and XGB. These models are integrated using a weight vector that assigns each model’s contribution to the final prediction accuracy. Optimizing these weights is crucial for improving the model’s generalization capability. To achieve this, the proposed IWMRFO is employed to identify the most effective weights, thereby increasing the accuracy of SI predictions. Essentially, this approach can be formulated as a mathematical equation composed of variables and coefficients: coefficients stand for the fixed predictions of each base model, and the variables represent the weights optimized by the proposed algorithm. The following equation describes the mathematical model of our approach:

23 $$\:\overrightarrow{{P}_{F}}={w}_{1}{\overrightarrow{x}}_{1}+{w}_{2}{\overrightarrow{x}}_{2}+{w}_{3}{\overrightarrow{x}}_{3}$$

where $\:{w}_{1}$, $\:{w}_{2}$, and $\:{w}_{3}$ represent the weights assigned to the predictions of the ET, HR, and XGB models, respectively, and $\:{\overrightarrow{x}}_{1}$, $\:{\overrightarrow{x}}_{2}$, and $\:{\overrightarrow{x}}_{3}$ denote the forecasts/coefficients produced by these models.

This equation can include more than three models if it improves prediction accuracy. We rely on these three models because, after extensive testing, we found they are the most effective for better SI predictions. The variables, or weights, in this equation are optimized using the proposed IWMRFO algorithm. Initially, the SI dataset undergoes preprocessing and is split into 80% training data and the remaining samples for testing. Each base ML model is then trained independently. Afterward, IWMRFO explores the search space to find the best combination of these models, aiming to improve overall generalization.

**Fig. 2:**
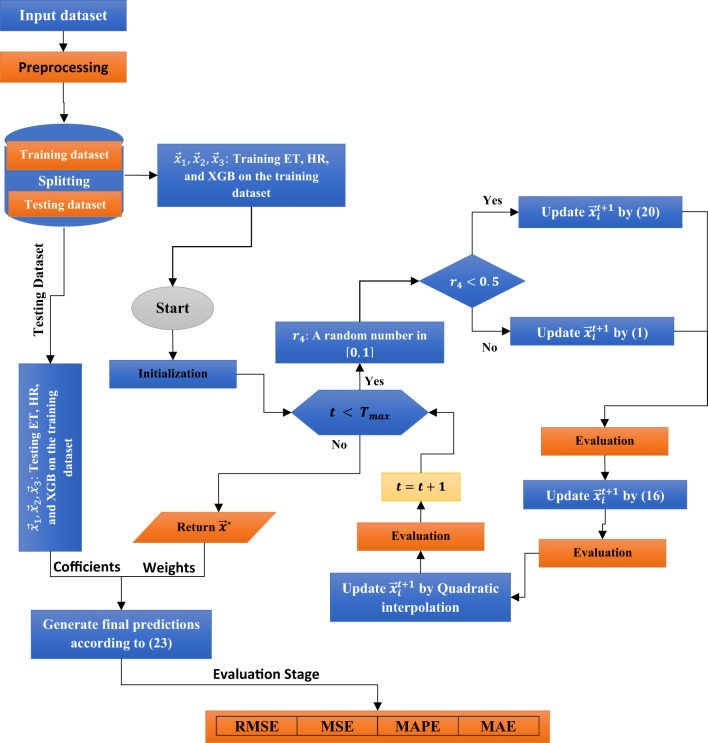
Flowchart of the proposed SIFA model.

The flowchart of the proposed SIFA framework is shown in Fig. 2. In this figure, the input SI dataset is initially preprocessed using normalization to improve learning. Afterward, feature selection is performed to identify the most informative features, which could improve prediction accuracy. The dataset is then split into training and testing sets, with the training data used to train the three main ML models: HR, XGB, and ET. The trained models are then input to the IWMRFO, which begins its optimization process by creating an $\:N\:\times\:\:D$ matrix. Each solution in this matrix is randomly initialized within the lower and upper bounds of the various ML components, as described in the experiments section. These solutions are then substituted into (23) to produce predicted values for the dataset samples. These predictions are evaluated by comparing them to the actual values using the MSE objective function defined below:

24 $$\:MSE=\frac{1}{n}\sum\:_{i=1}^{n}{({Y}_{i}-\stackrel{\sim}{{Y}_{i}})}^{2}$$

where $\:n$ stands for the number of samples, $\:{Y}_{i}$ is the estimated value for the $\:\mathrm{i}\mathrm{t}\mathrm{h}$ sample, and $\:\stackrel{\sim}{{Y}_{i}}$ represents the actual value for this sample.

The MSE values of different solutions within the population are compared, and the solution with the lowest MSE is identified as the best-so-far. This solution subsequently guides the optimization process to improve the quality of the remaining solutions. IWMRFO begins by iteratively updating candidate solutions within the population to generate new ones. Each new solution is substituted into (23) to produce predictions for the dataset samples. These predictions are then evaluated using the MSE objective function to determine each candidate solution’s fitness value. The fitness values are used to update the best-so-far solution whenever a better candidate is found. The IWMRFO optimization continues until $\:{T}_{\mathrm{m}\mathrm{a}\mathrm{x}}$ is reached. After completing the process, the best-so-far solution is returned and substituted into (23). This equation is then used to test the model’s ability to generalize. Each base model makes predictions on the testing dataset, which are assigned to their respective variables in (23) to produce the final predictions. These predictions are evaluated using multiple performance metrics, such as RMSE, MSE, R², and others discussed later.

## Experimental settings

In this study, the proposed model is evaluated using two popular datasets recorded at 24-hour intervals at two different locations: San Diego (DS1) and Islamabad (DS2), spanning from 2016 to 2021 and 2010 to 2021, respectively. In addition, the NASA SI (DS3) dataset, which includes columns such as wind direction, wind speed, humidity, and temperature along with solar radiation as the response parameter, is used to further evaluate the model performance. The three solved datasets include several features, some of which may be detrimental to the model’s performance; therefore, a feature selection technique is applied to eliminate those unbeneficial features. Before feature selection, they are standardized using the standard scaler defined in (25). This ensures that all features share the same scale, reducing bias toward dominant features and enhancing learning. 80% of each dataset is used for training the models, while the remaining 20% is used for testing. The proposed and compared models are trained and tested independently 10 times using random seeds ranging from 1 to 10 to ensure robust performance and reduce the risk of overfitting. Five well-known performance metrics—MAPE, RMSE, MBE, MAE, and R^2^—are used to evaluate the model’s performance. These metrics are extensively described in the next section. To assess the effectiveness and efficiency of the proposed SIFA, it is compared to several recent models, such as Parallel Boosting Neural Network (PBNN)^6^, ET Regression^9^, Random Forest (RF)^66^, Gaussian Process Regression (GPR)^67^, LightGBM^68^, LSTM^6^, BiLSTM-AADC^69^, and ANN^69^. The hyperparameters of these models are set according to the recommendations in the cited references to ensure a fair comparison. For the hyperparameters of the baseline models (HR, XGB, and ET), we rely on the default configurations provided by the scikit-learn library to maintain computational efficiency, as optimizing all baseline models’ hyperparameters would be too computationally costly. The comparison is based on the aforementioned performance metrics after running each algorithm 10 times independently. The resulting values for each metric are analyzed using six statistical measures: Best, Average (Avg), Worst, Standard Deviation (SD), P-value (PV) returned by the Wilcoxon signed-rank (WSR) test, and Friedman mean rank (FK). The WSR test is used to evaluate the statistical significance of the proposed model over the compared models. It is based on two hypotheses: null and alternative. The null hypothesis assumes that there is no significant difference between the results of the proposed and compared models, indicating that the new model does not provide a meaningful improvement. This hypothesis is accepted when the PV is greater than the significance level (typically 0.05). In contrast, the alternative hypothesis assumes that there is a statistically significant difference between the outcomes of the proposed and rival models, suggesting it may offer new contributions compared to existing ones. This hypothesis is achieved when the PV is less than 0.05. However, the PV does not capture the magnitude of the difference. As a result, to provide a standardized measure of the strength of the observed difference, the effect size ($\:Z/\surd\:N$) is employed and computed using the standardized Z-score obtained from the WSR test divided by the square root of the number of non-zero pairs $\:\left(N\right)$. Cohen’s criteria state that an effect is deemed insignificant if it is less than 0.1, small if it is between 0.1 and 0.3, medium if it is between 0.3 and 0.5, and large if it is greater than 0.5.

To observe the effectiveness of the proposed IWMRFO, it is compared to several rival algorithms, such as Manta Ray Foraging Optimization (MRFO)^54^, Artificial Gorilla Troops Optimization (AGTO)^70^, Gradient-Based Optimizer (GBO)^71^, Runge-Kutta optimizer (RUN)^72^, Artificial rabbits optimization (ARO)^73^, Energy Valley Optimizer (EVO)^74^, Giant Trevally Optimizer (GTO)^75^, Chernobyl Disaster Optimizer (CDO)^76^, Wavelet Mutation and Quadratic Interpolation MRFO (WMRFO)^55^. All algorithms’ controlling parameters are set as specified in the cited papers, except for $\:{T}_{max}$ and $\:N$, which are set to 200 and 25, respectively, to ensure a fair comparison. All ML models are built using hyperparameters recommended in the cited papers, employing Python 3.10, Keras for DL^77^, Mealpy for metaheuristic algorithms^78^, and scikit-learn for ML^79^. The device used for this research has an Intel Core i7 processor at 2.40 GHz and 32 GB of RAM.

25 $$\:{x}_{i}^{{\prime\:}}=\frac{{x}_{i}-\stackrel{-}{x}}{{a}_{i}}$$

where $\:{x}_{i}^{{\prime\:}}$ represents the $\:ith$ feature’s mean, $\:{x}_{i}$ refers to the value of the $\:ith$ feature, and $\:a$ is the SD of the $\:ith$ feature.

### Performance metrics

Five popular performance metrics—MAPE, RMSE, MAE, MSE, and R^2^—are used to evaluate the performance of both the proposed and rival models by measuring the differences between estimated and actual predictions, demonstrating each model’s effectiveness in addressing the solar irradiance problem. The metrics MAPE, RMSE, and MAE quantify the magnitude of prediction errors, whereas R² shows how effectively the predictions reflect the overall variability in the data. These metrics are mathematically defined as follows^80^:

26 $$\:\mathrm{R}\mathrm{M}\mathrm{S}\mathrm{E}=\sqrt{\frac{1}{n}\sum\:_{i=1}^{n}{({Y}_{i}-\stackrel{\sim}{{Y}_{i}})}^{2}}$$

27 $$\:MAE=\frac{1}{n}\:{\sum\:}_{i=1}^{n}\left|{Y}_{i}-\stackrel{\sim}{{Y}_{i}}\right|$$

28 $$\:MAPE=100\frac{1}{n}\:{\sum\:}_{i=1}^{n}\frac{\left|{Y}_{i}-\stackrel{\sim}{{Y}_{i}}\right|}{{Y}_{i}}$$

29 $$\:{R}^{2}=1-\frac{{\sum\:}_{i=1}^{n}{({Y}_{i}-\stackrel{\sim}{{Y}_{i}})}^{2}}{{\sum\:}_{i=1}^{n}{({Y}_{i}-\stackrel{-}{Y})}^{2}}$$

where $\:\stackrel{-}{Y}$ represents the mean of the actual values for all samples. All these metrics, except R², should be minimized, whereas R² should be maximized.

### Determination of optimal feature subset size

**Fig. 3:**
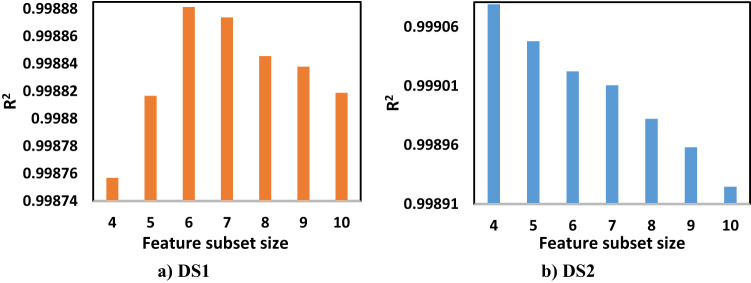
Performance evaluation versus number of features in the subset selected by SFS with RF.

The size of the feature subset selected by SFS is a hyperparameter that must be carefully tuned to enhance model performance. Therefore, various sizes, including 6, 7, 4, 5, 8, 9, and 10, are evaluated, and their results are shown in Fig. 3 for DS1 and DS2. Inspecting this figure shows that the proposed model can achieve significantly better accuracy on DS1 when the subset size is set to either 6 or 7, while for DS2, the best performance is obtained when it is set to 4. Accordingly, in the next experiments, we set this parameter to 6 for DS1 and 4 for DS2, as these values yield the best overall performance.

### Hyperparameter tuning of various ML components in SIFA

**Fig. 4:**
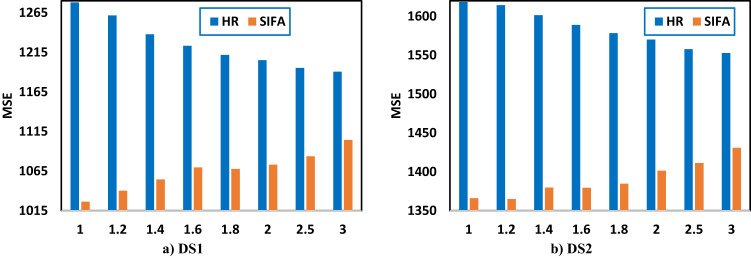
Hyperparameter tuning of Epsilon in HR.

**Fig. 5:**
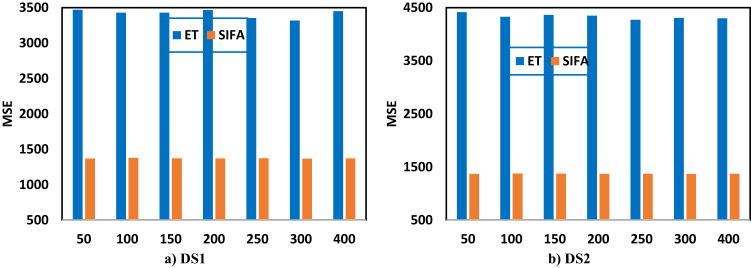
Hyperparameter tuning of n_estimators in ET.

**Fig. 6:**
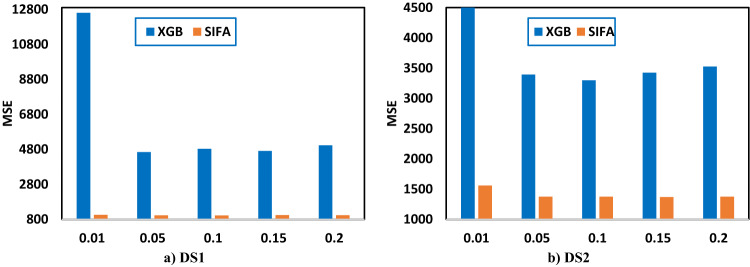
Hyperparameter tuning of Learning rate in XGB.

**Fig. 7:**
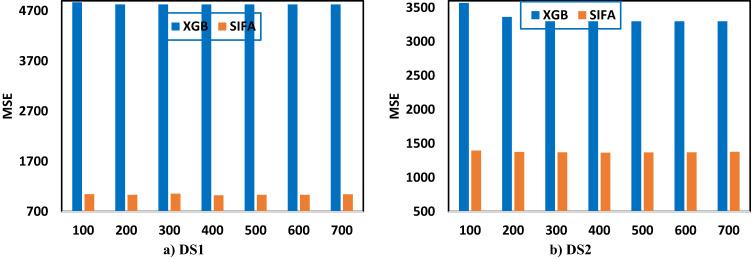
Hyperparameter tuning of n_estimators in XGB.

The proposed SIFA includes three ML models (HR, ET, and XGB), each with hyperparameters that can impact its training and testing performance. Therefore, we conduct extensive experiments to identify the best values for the most influential hyperparameters in each model. For instance, the HR model involves the Epsilon hyperparameter, with values greater than 1, which is believed to be crucial for enhancing generalization. To assess its impact, we test various Epsilon values (1, 1.2, 1.4, 1.6, 1.8, 2, 2.5, and 3) and analyze their effect on the baseline HR model individually as well as on its role within the proposed SIFA. The experimental results on DS1 and DS2 shown in Fig. 4 lead to two main observations. First, increasing Epsilon improves the HR model’s performance but causes the performance of the SIFA model to decline. Second, SIFA performs significantly better than the baseline HR model, indicating that the additional ML components integrated into SIFA enhance its generalization capabilities. Several additional hyperparameters, such as alpha and max_iter, in the HR have been evaluated under different values. However, experimental results showed that varying these parameters did not lead to significant changes in model performance. Therefore, the default values provided by the scikit-learn library were retained. Likewise, we tune the number of estimators in the ET model and examine the influence of various values on the ET model individually and on the proposed SIFA model, as reported in Fig. 5. The results indicate that the performance of ET and SIFA is not significantly affected by varying the number of estimators. Therefore, the default value provided by the SciKit-Learn library is retained. The XGB model includes two key hyperparameters—the learning rate and the number of estimators—that need to be tuned to achieve optimal performance. Accordingly, several experiments are conducted using different values for each parameter, and the results on DS1 and DS2 are shown in Figs. 6 and 7. The experimental results show that low learning rates (below 0.01) lead to slower convergence and inferior performance, whereas higher rates yield better results on both datasets. For learning rates above 0.01, performance is relatively stable; therefore, we use 0.1 for subsequent experiments. Regarding the number of estimators (Fig. 7), the results show comparable performance across all tested values. Furthermore, the experimental results demonstrate that the proposed SIFA is significantly better than XGB across all tested values of both hyperparameters. Overall, we conclude from the results reported in this section that SIFA is significantly better than three baseline ML components (HR, XGB, and ET) used in its construction, highlighting its superiority in enhancing generalization.

### Determination of upper bounds for each component in the proposed SIFA

**Fig. 8:**
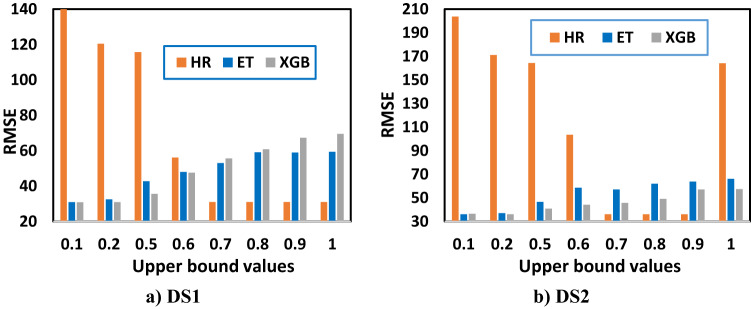
Performance evaluation under different upper-bound values on DS1 and DS2.

Applying the same upper bounds to all components might bias the optimization process toward certain models, even if their generalization performance is poor. Consequently, some components could reach high training accuracy but perform poorly on unseen test data. Thus, selecting the appropriate upper bound for each component can enhance the performance of the proposed IWMRFO and help find near-optimal values that minimize overfitting while ensuring strong predictive performance on both training and testing datasets. Therefore, in this section, the upper bound for each ML component within the proposed SIFA model is explored by evaluating a range of values (0.1, 0.2, 0.5, 0.6, 0.7, 0.8, 0.9, and 1.0) on both DS1 and DS2. In these experiments, we consider RMSE as a performance metric to observe effectiveness, with results reported on both datasets in Fig. 8. This figure shows that the performance of SIFA is significantly improved when setting the upper bounds of HR, ET, and XGB to 0.8, 0.1, and 0.1, respectively. This indicates that this hyperparameter has a notable effect on generalization performance, and that carefully tuning it can lead to more accurate results.

## Results and discussions

This section reports the results of the proposed SIFA model on three popular datasets. It also compares these results with several competing models using performance metrics such as RMSE, MSE, MAPE, MAE, and R², demonstrating its effectiveness in predicting SI. Additionally, this section evaluates the effectiveness of the proposed IWMRFO against the standard WMRFO and other algorithms to assess if the integration of chaotic maps improves outcomes. Furthermore, we examine various chaotic maps to identify which ones deliver better results.

### Performance evaluation of different chaotic maps

**Fig. 9:**
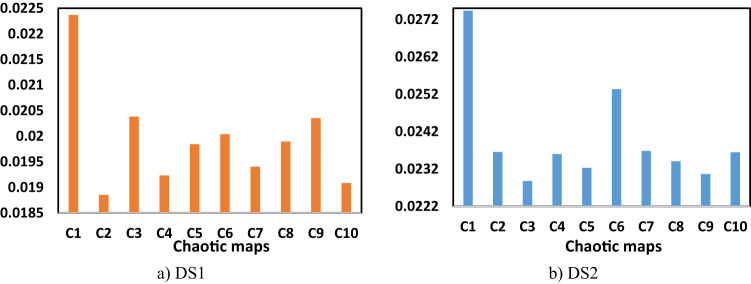
Results of ten different chaotic maps on both SI datasets.

The proposed IWMRFO uses chaotic maps to boost its exploration capabilities when searching for optimal weights of various ML models in the SIFA model. Recognizing that different chaotic maps can influence model performance in different ways, this section tests ten chaotic maps listed in Table 1 to find out which one yields the best IWMRFO results. The impact of each map on DS1 and DS2 is shown in Fig. 9. Clearly, C2 performs best on DS1, followed by C10 and C4, while C1 is the least effective. On DS2, C3 slightly beats C9 and significantly outperforms the other maps. Therefore, for the next experiments, C2 was selected for DS1 and C3 for DS2 because of their superior ability to improve IWMRFO.

### Comparison between IWMRFO and some recent metaheuristic algorithms

**Fig. 10:**
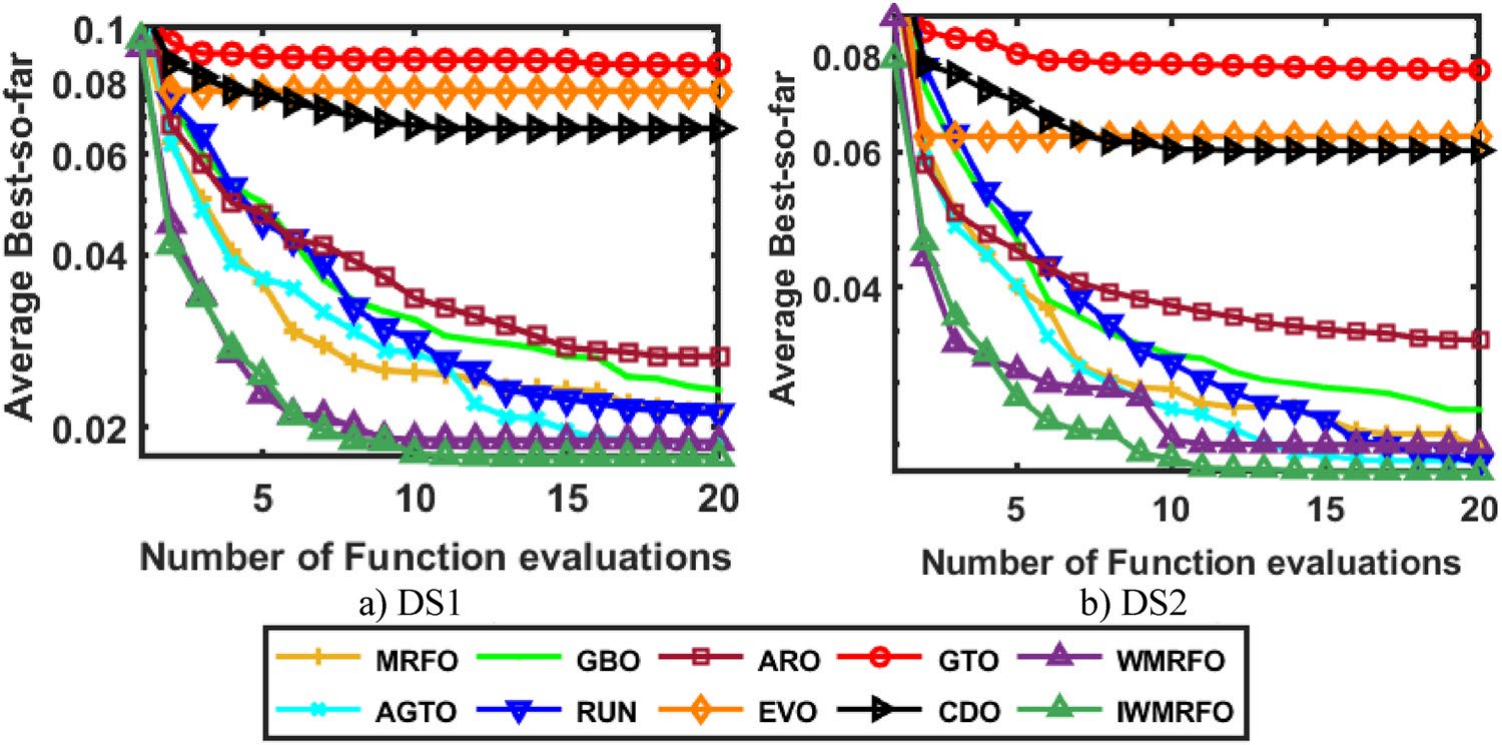
Convergence curves of the proposed IWMRFO and rival algorithms on both SI datasets.

In this section, the performance of the proposed IWMRFO is compared to nine recent competitors using various statistical measures to highlight its effectiveness in detecting each base model’s contribution within the proposed SIFA. On each dataset, each algorithm is run 15 independent times, and the resulting fitness values are analyzed using the best, average, worst, SD, and FK metrics, as shown in Table 2. This table shows that the proposed algorithm is competitive with some algorithms regarding the best value and outperforms all others on the remaining performance metrics. Overall, in terms of the FK value, IWMRFO achieves 2.47 for DS1 and DS2, followed by AGTO with 2.77 for DS1 and 2.57 for DS2. In contrast, GTO has the poorest rank, with 9.47 for DS1 and 9.87 for DS2. The WSR test is used to determine whether the improvement of IWMRFO is statistically significant. Table 2 shows the PVs between the outcomes of the proposed IWMRFO and those of the rival algorithms. The results demonstrate that for DS1, IWMRFO’s outcomes differ significantly from all the other algorithms. For DS2, it shows some similarity to algorithms like MRFO, AGTO, and WMRFO, while differing considerably from the rest. To illustrate the convergence speed of the algorithms during optimization, Fig. 10 presents their convergence curves on DS1 and DS2. The figure indicates that IWMRFO exhibits robust exploitation capabilities, allowing it to achieve rapid initial convergence. Moreover, it effectively avoids local optima throughout the process, as demonstrated by the consistent downward trend.

**Table 2:** Results of the proposed IWMRFO and nine competing metaheuristic algorithms on DS1 and DS2.

Datasets		MRFO	AGTO	GBO	RUN	ARO	EVO	GTO	CDO	WMRFO	IWMRFO
**DS1**	**Best**	**1.7704E-02**	**1.7704E-02**	1.7705E-02	**1.7704E-02**	1.7705E-02	5.1410E-02	7.2122E-02	1.9756E-02	**1.7704E-02**	**1.7704E-02**
	**Avg**	2.1328E-02	1.8828E-02	2.2890E-02	2.1087E-02	2.6372E-02	7.7637E-02	8.6366E-02	6.6712E-02	1.8824E-02	**1.7707E-02**
	**Wrst**	2.7564E-02	2.7046E-02	2.9381E-02	2.6718E-02	5.4746E-02	9.6011E-02	9.1916E-02	8.9734E-02	2.7046E-02	**1.7739E-02**
	**SD**	4.6084E-03	2.9772E-03	4.7232E-03	3.8971E-03	1.0064E-02	1.2975E-02	6.5634E-03	1.9892E-02	2.6926E-03	**8.8738E-06**
	**PV**	**4.88E-02**	**4.09E-02**	**5.04E-06**	**8.12E-05**	**5.23E-05**	**3.38E-06**	**3.38E-06**	**3.38E-06**	**2.67E-02**	
	**FK**	4.07	2.77	5.60	4.73	5.67	9.07	9.47	8.20	2.97	**2.47**
**DS2**	**Best**	**2.2220E-02**	**2.2220E-02**	**2.2220E-02**	**2.2220E-02**	2.2297E-02	4.5116E-02	6.3063E-02	2.3830E-02	**2.2220E-02**	**2.2220E-02**
	**Avg**	2.4523E-02	2.3783E-02	2.7428E-02	2.3451E-02	3.3843E-02	6.2972E-02	7.6749E-02	6.0355E-02	2.4912E-02	**2.3002E-02**
	**Wrst**	**3.3941E-02**	**3.3941E-02**	6.5294E-02	2.8951E-02	5.6531E-02	7.7370E-02	8.2394E-02	8.5612E-02	3.3941E-02	**3.3941E-02**
	**SD**	4.5220E-03	4.1240E-03	1.0977E-02	**2.2256E-03**	9.1350E-03	1.0041E-02	5.0254E-03	1.5229E-02	4.7356E-03	3.0263E-03
	**PV**	1.71E-01	4.76E-01	**3.70E-05**	**6.39E-05**	**9.72E-06**	**1.90E-06**	**1.90E-06**	**2.35E-06**	5.22E-01	
	**FK**	3.33	2.57	5.47	4.67	6.40	8.60	9.87	8.20	3.43	**2.47**

**Bold values** represent the best findings.

### Performance evaluation of SFS with RF

This section examines the effectiveness of the SFS method when used with RF. We compare it against other FS techniques, such as SelectFromModel (SFM) with Lasso regression, the mutual information criterion, and the RF-based Recursive Feature Elimination (RFE) technique^13^. Moreover, we assess the entire feature set to determine whether FS provides a significant advantage. On both datasets, the feature subsets extracted by SFS, SFM, RFE, and MI, along with the original feature set, are evaluated, and the corresponding performance metrics are reported in Table 3. This table shows that SFS can identify the feature subset that maximizes model performance across five performance metrics. For example, on DS2, RMSE was 55.26 with SFS, 59.22 with RFE, 59.36 with SFM, 59.59 with MI, and 61.70 with the complete feature set. This demonstrates the high effectiveness of SFS combined with RF, as it can approximate an optimal feature subset that significantly enhances model performance, with an improvement percentage of up to approximately 7%.

**Table 3:** Results of SFS and other FS techniques.

		Islamada (DS2)					San Diego (DS1)			
	SFS	RFE	SFM	MI	Standard	SFS	RFE	SFM	MI	Standard
**MSE**	**3055.56083**	3508.78052	3526.37036	3553.02695	3809.29424	**2731.03909**	3203.59852	3939.12552	3247.25123	3374.23411
**RMSE**	**55.2614154**	59.2251357	59.3650630	59.5904709	61.7042925	**52.1938897**	56.5617270	62.7290175	56.9281352	58.0213227
**MAE**	**40.6790976**	44.0170065	44.4364073	44.6687029	46.3359404	**39.4619352**	42.9542829	48.0780420	42.9929723	44.2621621
**MAPE**	**0.97193532**	1.02504277	1.05959885	1.06391196	1.10438086	**0.87102892**	0.93675081	1.06272199	0.96466963	0.99931604
**R** ^**2**^	**0.99908158**	0.99894519	0.99893994	0.99893203	0.99885505	**0.9989955**	0.99882103	0.99854907	0.99880456	0.99875759

**Bold values** represent the best findings.

### Proposed component contribution and comparison with stacking and voting regressors

**Fig. 11:**
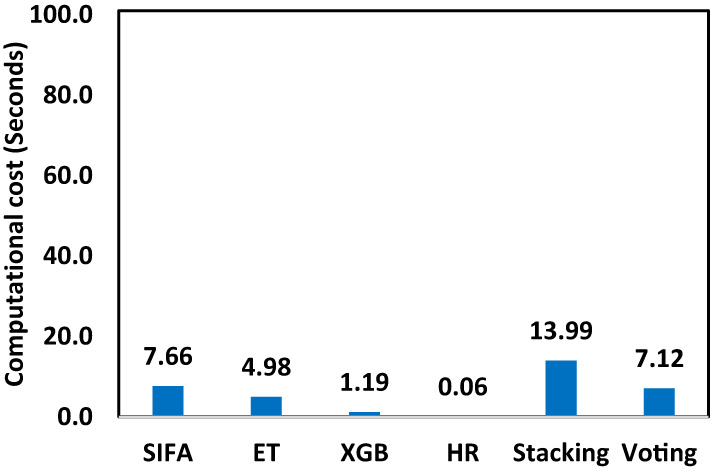
Training time of proposed SIFA components vs. stacking and voting Models.

**Fig. 12:**
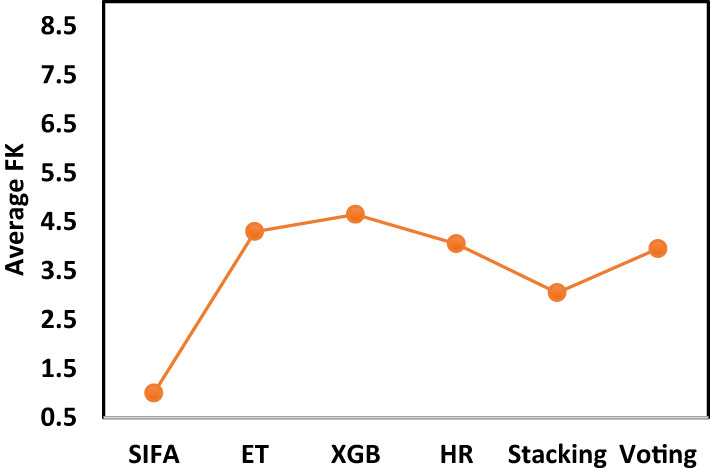
Average FK of proposed SIFA components vs. stacking and voting Models.

In this section, we analyze the effectiveness of the individual components of the proposed SIFA framework and demonstrate how the proposed IWMRFO method efficiently ensembles three base models (XGB, HR, and ET). We further compare its performance with other ensemble techniques, including voting and stacking regressors. For the voting regressor, the same base models are used as estimators, whereas in the stacking model, XGB and ET are employed as base learners, with HR serving as the meta-learner. The average results of various proposed components individually, in addition to the other ensemble methods on the DS2 and DS3 datasets, are shown in Table 4. These results emphasize the effectiveness of the SIFA model over all individual components and ensemble methods, indicating that employing IWMRFO to derive each base model’s contribution can lead to maximizing the SI prediction accuracy. In addition, this table shows the FK values of various models to illustrate the significant improvement of the proposed model over each compared model. These values show that it is significantly better than all others on both datasets, followed by the stacking classifier, while XGB performs the worst on DS2 and HR on DS3. To further highlight the significance of the SIFA improvements, the p-value returned by the WSR test is shown in the same table, which indicates that it could achieve results significantly different from those of the other compared models. To show the overall performance of various models, the average FK on both datasets is presented in Fig. 11, which confirms our previous analysis that SIFA is the best, the stacking regressor is the second best, and ET and XGB are the least effective. Finally, the computational cost of various models is reported in Fig. 12, which shows that SIFA is slower than the individual components but is competitive with stacking and voting regressors.

**Table 4:** SIFA component results and comparison with stacking and voting.

		DS2						DS3					
Metrics		SIFA	ET	XGB	HR	Stacking	Voting	SIFA	ET	XGB	HR	Stacking	Voting
**MSE**	Avg	**1300.47**	4400.56	3298.75	1570.08	2793.52	1788.34	**6254.25**	6398.11	6730.14	37298.27	6324.51	9750.11
	PV		**2.0E-03**	**2.0E-03**	**2.0E-03**	**2.0E-03**	**2.0E-03**		**1.9E-03**	**1.9E-03**	**1.9E-03**	**1.9E-03**	**1.9E-03**
	FK	**1.00**	5.70	5.30	2.10	4.00	2.90	**1.00**	2.90	4.00	6.00	2.10	5.00
**RMSE**	Avg	**36.062**	66.336	57.435	39.624	52.854	42.288	**79.073**	79.979	82.027	193.125	79.515	98.736
	PV		**2.0E-03**	**2.0E-03**	**2.0E-03**	**2.0E-03**	**2.0E-03**		**1.9E-03**	**1.9E-03**	**1.9E-03**	**1.9E-03**	**1.9E-03**
	FK	**1.00**	5.70	5.30	2.10	4.00	2.90	**1.00**	2.90	4.00	6.00	2.10	5.00
**R** ^**2**^	Avg	**0.9996**	0.9987	0.9990	0.9995	0.9992	0.9995	**0.9373**	0.9359	0.9325	0.6260	0.9366	0.9022
	PV		**2.0E-03**	**2.0E-03**	**2.0E-03**	**2.0E-03**	**2.0E-03**		**1.9E-03**	**1.9E-03**	**1.9E-03**	**1.9E-03**	**1.9E-03**
	FK	**1.00**	5.70	5.30	2.10	4.00	2.90	**1.00**	2.90	4.00	6.00	2.10	5.00

*Bold values in the PV row indicate the significance of the SIFA improvements, while Bold values in the remaining rows stand for the best outcomes. All reported values are computed on the held-out test set.

### Performance evaluation over DS1

**Fig. 13:**
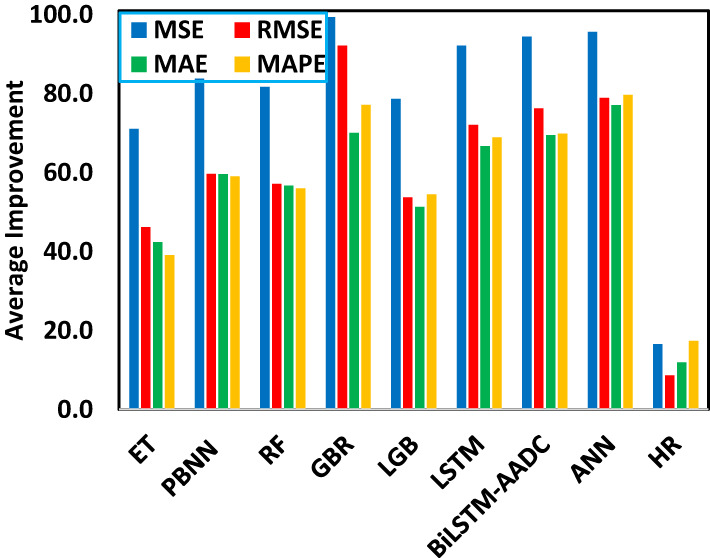
IR(%) of SIFA over rival models for DS1.

**Fig. 14:**
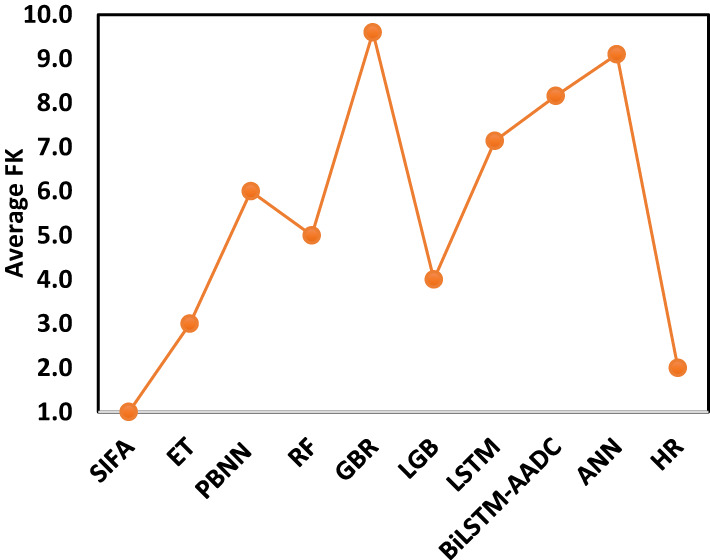
Average FK of various models on all performance metrics for DS1.

**Fig. 15:**
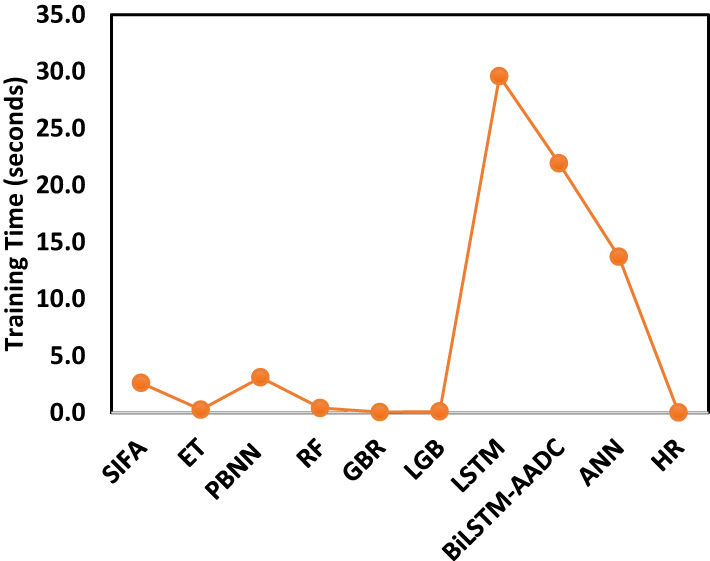
Training time of various models on DS1.

**Fig. 16:**
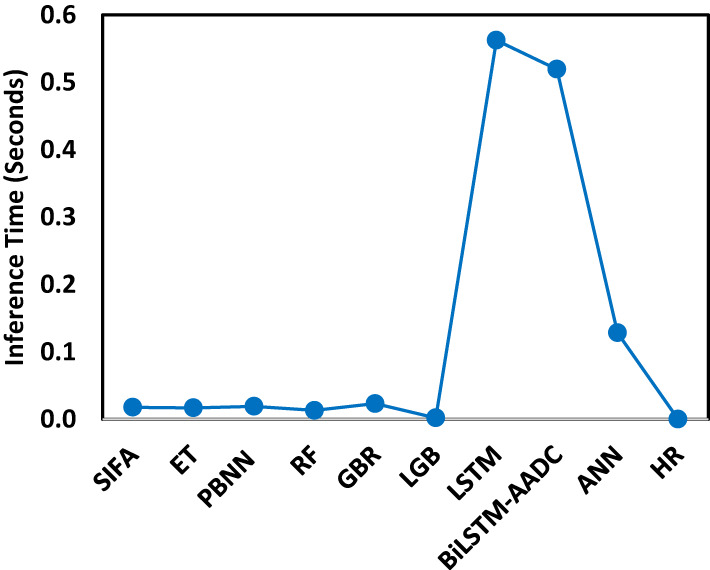
Inference time of various models on DS1.

This section presents the results of the proposed SIFA method and competing models for DS1, demonstrating their robustness in predicting solar irradiance. To evaluate the stability and resistance to overfitting, the training-testing process for each model is repeated 10 times independently. In each run, each model is trained on 80% of the entire dataset and subsequently evaluated on the remaining 20% using the specified performance metrics. The results for these metrics are analyzed with six statistical indicators and summarized in Table 5. The table shows that SIFA outperforms all other models across all metrics. Overall, SIFA achieves an FK value of 1 for all metrics, while HR ranks second, and GBR is the poorest. These findings are also confirmed in Fig. 13, which displays the average FK values across all performance metrics for each model. To quantify SIFA’s improvement over other models, the improvement rate (IR) metric is used. This additional metric measures the percentage improvement of SIFA relative to each comparator, as defined in (30). Figure 14 displays the IR for each model across different metrics, showing that the smallest improvement rate occurs with HR, with IRs ranging from 9.0% to 17.5%. This indicates that SIFA has outstanding characteristics that enable it to surpass several existing models in lowering error variability. To show the significance of the proposed models over the compared models, the PVs comparing SIFA with each model across all metrics are listed in Table 5. All PVs show that the null hypothesis can be rejected, confirming that the improvements are statistically significant. Finally, Figs. 15 and 16 illustrate the training and inference times for each model, demonstrating their training and testing speeds. These figures show that SIFA is slower than traditional ML models like RF, HR, GBR, LGB, and ET, but considerably faster than all the DL models compared. Its lower efficiency compared to ML models is due to its structure, which includes several key components that enable it to achieve outstanding prediction results. The first component is the use of IWMRFO to determine each base model’s contributions; the second is employing three different ML models to better learn nonlinearity in the SI data, with each model requiring separate training and testing before being combined using IWMRFO-derived weights. Despite this, its impressive and effective results still make it the best alternative for accurately predicting SI.

30 $$\:IR\:\left({\%}\right)=\frac{\left({E}_{c}-{E}_{p}\right)}{{E}_{c}}\times\:100$$

where $\:{E}_{c}$ is the result from a comparison model for each performance metric, and $\:{E}_{p}$ indicates the result from the proposed model.

**Table 5:** Results of the proposed SIFA and eight competing models on DS1.

Metrics		SIFA	ET	PBNN	RF	GBR	LGB	LSTM	BiLSTM-AADC	ANN	HR
**MSE**	Best	**9.42E + 02**	3.35E + 03	6.10E + 03	5.43E + 03	1.64E + 05	4.73E + 03	1.16E + 04	1.42E + 04	1.33E + 04	1.21E + 03
	Avg	**1.01E + 03**	3.49E + 03	6.22E + 03	5.51E + 03	1.64E + 05	4.73E + 03	1.29E + 04	1.81E + 04	2.34E + 04	1.21E + 03
	Worst	**1.03E + 03**	3.59E + 03	6.32E + 03	5.74E + 03	1.64E + 05	4.73E + 03	1.46E + 04	2.26E + 04	3.53E + 04	1.21E + 03
	SD	2.54E + 01	7.95E + 01	6.82E + 01	9.24E + 01	3.07E-11	0.00E + 00	1.04E + 03	2.46E + 03	7.60E + 03	**2.40E-13**
	PV		**0.0051**	**0.0051**	**0.0051**	**0.0051**	**0.0051**	**0.0051**	**0.0051**	**0.0051**	**0.0051**
	FK	**1.00**	3.00	6.00	5.00	10.00	4.00	7.00	8.30	8.70	2.00
**RMSE**	Best	**3.07E + 01**	5.79E + 01	7.81E + 01	7.37E + 01	4.05E + 02	6.87E + 01	1.08E + 02	1.19E + 02	1.15E + 02	3.48E + 01
	Avg	**3.18E + 01**	5.91E + 01	7.89E + 01	7.42E + 01	4.05E + 02	6.87E + 01	1.14E + 02	1.34E + 02	1.51E + 02	3.48E + 01
	Worst	**3.20E + 01**	5.99E + 01	7.95E + 01	7.58E + 01	4.05E + 02	6.87E + 01	1.21E + 02	1.50E + 02	1.88E + 02	3.48E + 01
	SD	4.06E-01	6.75E-01	4.32E-01	6.18E-01	5.99E-14	1.50E-14	4.57E + 00	9.28E + 00	2.52E + 01	**7.49E-15**
	PV		**0.0051**	**0.0051**	**0.0051**	**0.0051**	**0.0051**	**0.0051**	**0.0051**	**0.0051**	**0.0051**
	FK	**1.00**	3.00	6.00	5.00	10.00	4.00	7.00	8.30	8.70	2.00
**MAE**	Best	**2.36E + 01**	4.11E + 01	5.92E + 01	5.56E + 01	8.10E + 01	4.99E + 01	6.68E + 01	6.85E + 01	8.31E + 01	2.76E + 01
	Avg	**2.43E + 01**	4.22E + 01	6.02E + 01	5.61E + 01	8.10E + 01	4.99E + 01	7.30E + 01	7.95E + 01	1.06E + 02	2.76E + 01
	Worst	**2.45E + 01**	4.27E + 01	6.09E + 01	5.71E + 01	8.10E + 01	4.99E + 01	8.12E + 01	9.56E + 01	1.37E + 02	2.76E + 01
	SD	2.61E-01	4.85E-01	4.99E-01	4.68E-01	1.50E-14	7.49E-15	4.86E + 00	7.23E + 00	1.91E + 01	**3.74E-15**
	PV		0.0051	0.0051	0.0051	0.0051	0.0051	0.0051	0.0051	0.0051	0.0051
	FK	**1.00**	3.00	6.00	5.00	8.60	4.00	7.20	8.30	9.90	2.00
**MAPE**	Best	**5.32E-01**	8.60E-01	1.29E + 00	1.21E + 00	2.35E + 00	1.18E + 00	1.56E + 00	1.49E + 00	2.07E + 00	6.50E-01
	Avg	**5.37E-01**	8.82E-01	1.31E + 00	1.22E + 00	2.35E + 00	1.18E + 00	1.73E + 00	1.78E + 00	2.64E + 00	6.50E-01
	Worst	**5.53E-01**	8.95E-01	1.33E + 00	1.24E + 00	2.35E + 00	1.18E + 00	1.90E + 00	2.29E + 00	3.53E + 00	6.50E-01
	SD	5.93E-03	9.66E-03	1.10E-02	9.89E-03	**0.00E + 00**	2.34E-16	1.13E-01	2.05E-01	5.08E-01	1.17E-16
	PV		**0.0051**	**0.0051**	**0.0051**	**0.0051**	**0.0051**	**0.0051**	**0.0051**	**0.0051**	**0.0051**
	FK	**1.00**	3.00	6.00	5.00	9.40	4.00	7.50	7.60	9.50	2.00
**R** ^**2**^	Best	**0.99960**	0.99860	0.99753	0.99776	0.93579	0.99815	0.99431	0.99117	0.98619	0.99953
	Avg	**0.99960**	0.99864	0.99757	0.99785	0.93579	0.99815	0.99495	0.99291	0.99086	0.99953
	Worst	**0.99963**	0.99869	0.99762	0.99788	0.93579	0.99815	0.99547	0.99447	0.99479	0.99953
	SD	9.98E-06	3.09E-05	2.67E-05	3.62E-05	**0.00E + 00**	1.17E-16	4.07E-04	9.63E-04	2.97E-03	2.34E-16
	PV		**0.0051**	**0.0051**	**0.0050**	**0.0049**	**0.0049**	**0.0051**	**0.0051**	**0.0051**	**0.0049**
	FK	**1.00**	3.00	6.00	5.00	10.00	4.00	7.00	8.30	8.70	2.00

*Bold values in the PV row indicate the significance of the SIFA improvements, while Bold values in the remaining rows stand for the best outcomes. All reported values are computed on the held-out test set.

### Performance evaluation over DS2

**Fig. 17:**
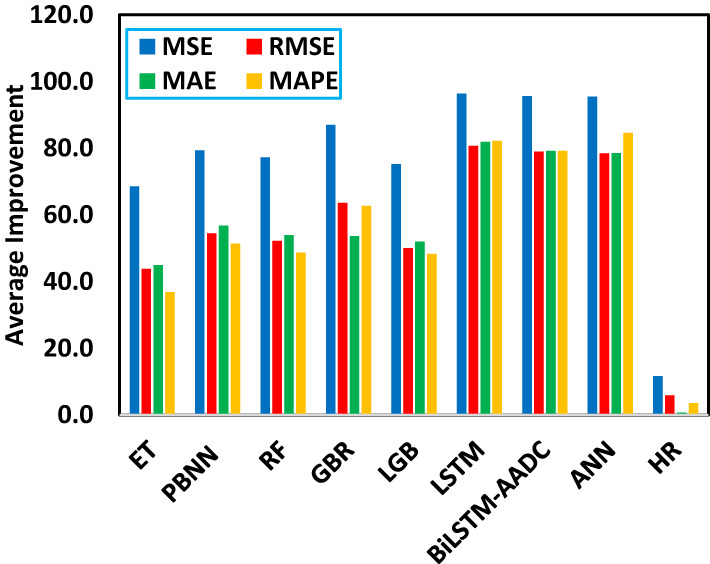
IR(%) of SIFA over rival models for DS2.

**Fig. 18:**
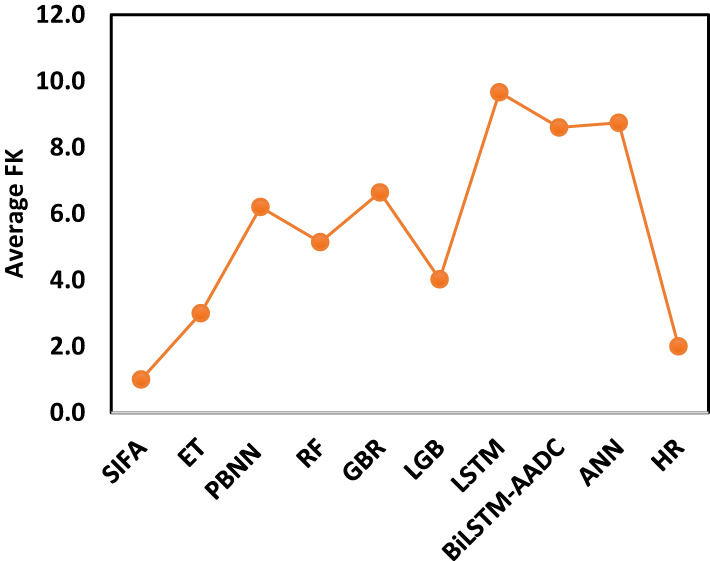
Average FK of various models on all performance metrics for DS2.

**Fig. 19:**
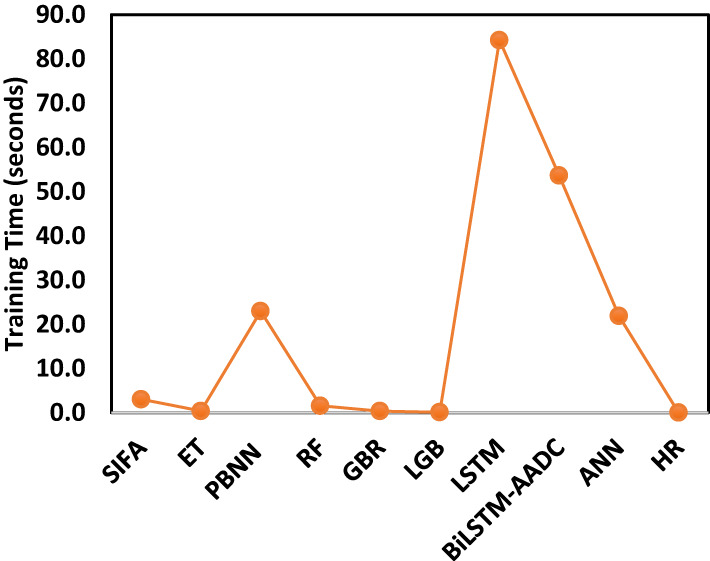
Training time of various models on DS2.

**Fig. 20:**
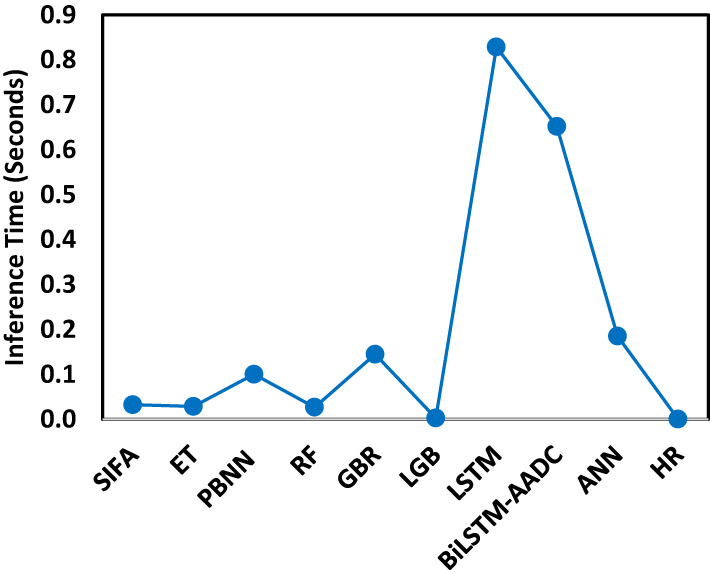
Inference time of various models on DS2.

This section shows the results of the proposed SIFA method alongside other models for DS2, emphasizing its strong capability to predict solar irradiance reliably. Similar to DS1, DS2 is divided into 80% for training and 20% for testing. The performance of various models is evaluated 10 independent times, and the obtained results are summarized with six statistical indicators and presented in Table 6. These results clearly demonstrate that SIFA consistently outperforms all other models across all performance metrics. Remarkably, SIFA achieves a perfect FK value of 1 across all metrics, followed by ET, while BiLSTM-AADC and ANN achieve the worst performance. These results are also confirmed in Fig. 17, which shows the average FK values for each model. To better understand SIFA’s superior performance, we used the IR metric, which assesses how much better SIFA performs compared to other models. Figure 18 displays these IR values across different metrics, with ET exhibiting the lowest IR for most performance measures, ranging from 1% to 12%. These findings highlight SIFA’s outstanding ability to surpass various existing models in predicting the SI. We also tested the significance of these improvements using the WSR test. The p-values from this test, comparing SIFA with each model across all metrics, are shown in Table 6 and support rejecting the null hypothesis, confirming that the improvements are statistically significant. Additionally, Figs. 19 and 20 display the training and inference times for each model, showing how quickly they can be trained and tested. These figures indicate that our proposed model is slower than traditional ML models such as RF, HR, GBR, LGB, and ET, but significantly faster than all the compared DL models.

**Table 6:** Results of the proposed SIFA and eight competing models on DS2.

Metrics		SIFA	ET	PBNN	RF	GBR	LGB	LSTM	BiLSTM-AADC	ANN	HR
**MSE**	Best	**1.37E + 03**	4.28E + 03	6.47E + 03	5.92E + 03	1.05E + 04	5.52E + 03	2.99E + 04	2.71E + 04	2.22E + 04	1.55E + 03
	Avg	**1.37E + 03**	4.36E + 03	6.63E + 03	6.02E + 03	1.05E + 04	5.52E + 03	3.73E + 04	3.11E + 04	2.99E + 04	1.55E + 03
	Worst	**1.38E + 03**	4.44E + 03	6.81E + 03	6.10E + 03	1.05E + 04	5.52E + 03	5.73E + 04	3.45E + 04	3.47E + 04	1.55E + 03
	SD	2.88E + 00	5.20E + 01	1.27E + 02	5.31E + 01	1.92E-12	**0.00E + 00**	7.79E + 03	2.52E + 03	4.09E + 03	2.40E-13
	PV		**0.0051**	**0.0051**	**0.0051**	**0.0051**	**0.0051**	**0.0051**	**0.0051**	**0.0051**	**0.0051**
	FK	**1.00**	3.00	6.00	5.00	7.00	4.00	9.80	8.70	8.50	2.00
**RMSE**	Best	**3.70E + 01**	6.55E + 01	8.04E + 01	7.69E + 01	1.02E + 02	7.43E + 01	1.73E + 02	1.64E + 02	1.49E + 02	3.94E + 01
	Avg	**3.71E + 01**	6.60E + 01	8.14E + 01	7.76E + 01	1.02E + 02	7.43E + 01	1.92E + 02	1.76E + 02	1.72E + 02	3.94E + 01
	Worst	**3.71E + 01**	6.66E + 01	8.25E + 01	7.81E + 01	1.02E + 02	7.43E + 01	2.39E + 02	1.86E + 02	1.86E + 02	3.94E + 01
	SD	3.89E-02	3.94E-01	7.77E-01	3.43E-01	**0.00E + 00**	1.50E-14	1.88E + 01	7.21E + 00	1.22E + 01	0.00E + 00
	PV		**0.0051**	**0.0051**	**0.0051**	**0.0051**	**0.0051**	**0.0051**	**0.0051**	**0.0051**	**0.0051**
	FK	**1.00**	3.00	6.00	5.00	7.00	4.00	9.80	8.70	8.50	2.00
**MAE**	Best	**2.67E + 01**	4.82E + 01	6.09E + 01	5.72E + 01	5.76E + 01	5.56E + 01	1.33E + 02	1.19E + 02	1.09E + 02	2.69E + 01
	Avg	**2.67E + 01**	4.85E + 01	6.18E + 01	5.79E + 01	5.76E + 01	5.56E + 01	1.47E + 02	1.28E + 02	1.24E + 02	2.69E + 01
	Worst	**2.68E + 01**	4.89E + 01	6.27E + 01	5.82E + 01	5.76E + 01	5.56E + 01	1.78E + 02	1.35E + 02	1.33E + 02	2.69E + 01
	SD	4.23E-02	2.40E-01	5.95E-01	3.38E-01	**0.00E + 00**	**0.00E + 00**	1.35E + 01	5.47E + 00	7.88E + 00	3.74E-15
	PV		**0.0051**	**0.0051**	**0.0051**	**0.0051**	**0.0051**	**0.0051**	**0.0051**	**0.0051**	**0.0051**
	FK	**1.00**	3.00	7.00	5.80	5.20	4.00	9.90	8.80	8.30	2.00
**MAPE**	Best	**7.37E-01**	1.16E + 00	1.49E + 00	1.43E + 00	1.98E + 00	1.43E + 00	3.66E + 00	3.26E + 00	4.12E + 00	7.66E-01
	Avg	**7.39E-01**	1.17E + 00	1.52E + 00	1.44E + 00	1.98E + 00	1.43E + 00	4.15E + 00	3.56E + 00	4.78E + 00	7.66E-01
	Worst	**7.40E-01**	1.19E + 00	1.55E + 00	1.45E + 00	1.98E + 00	1.43E + 00	5.92E + 00	3.78E + 00	5.38E + 00	7.66E-01
	SD	9.46E-04	7.54E-03	2.11E-02	7.68E-03	**0.00E + 00**	2.34E-16	6.63E-01	1.78E-01	3.69E-01	1.17E-16
	PV		**0.0051**	**0.0051**	**0.0051**	**0.0051**	**0.0051**	**0.0051**	**0.0051**	**0.0051**	**0.0051**
	FK	**1.00**	3.00	6.00	4.90	7.00	4.10	9.00	8.10	9.90	2.00
**R** ^**2**^	Best	**0.99958**	0.99866	0.99794	0.99816	0.99683	0.99833	0.98267	0.98957	0.98951	0.99953
	Avg	**0.99958**	0.99868	0.99800	0.99818	0.99683	0.99833	0.98872	0.99059	0.99097	0.99953
	Worst	**0.99959**	0.99871	0.99804	0.99821	0.99683	0.99833	0.99097	0.99182	0.99328	0.99953
	SD	9.19E-07	1.57E-05	3.82E-05	1.59E-05	1.17E-16	1.17E-16	2.36E-03	7.60E-04	1.24E-03	**0.00E + 00**
	PV		0.0051	0.0051	0.0050	0.0045	0.0045	0.0051	0.0051	0.0051	0.0045
	FK	**1.00**	3.00	6.00	5.00	7.00	4.00	9.80	8.70	8.50	2.00

*Bold values in the PV row indicate the significance of the SIFA improvements, while Bold values in the remaining rows stand for the best outcomes. All reported values are computed on the held-out test set.

### Performance evaluation over DS3

**Fig. 21:**
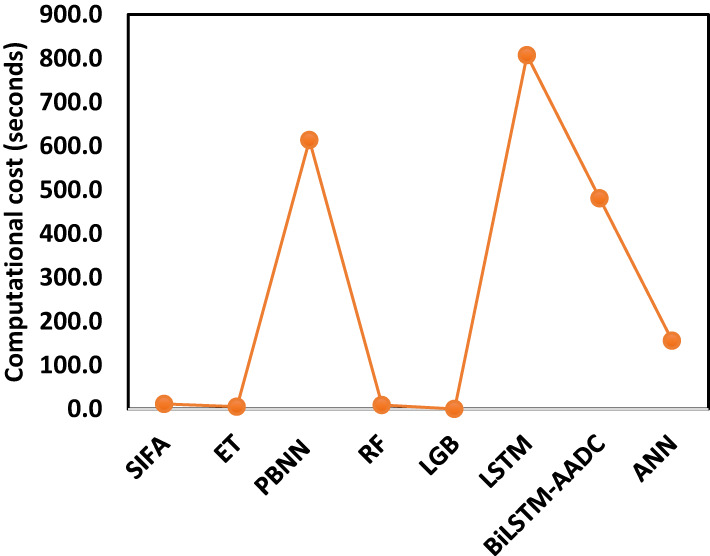
Average Computational cost on DS3.

**Fig. 22:**
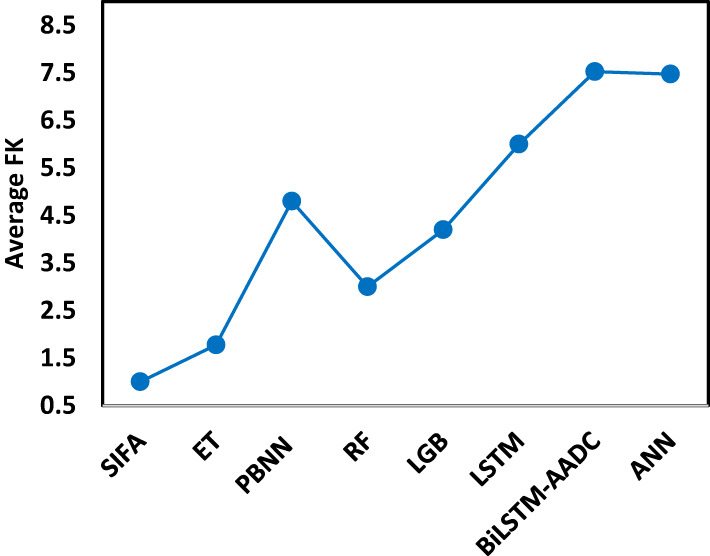
Average FK of various models on all performance metrics for DS3.

In this section, we evaluate the performance of the proposed and competing models on DS3 to further assess training performance and generalization capability. Similar to the previous datasets, the dataset is divided into training and testing sets with an 80:20 split ratio. Each model is trained independently 10 times. The results are analyzed using the aforementioned evaluation metrics and reported in Table 7. As shown in this table, SIFA outperforms all compared models across all evaluation metrics. Specifically, based on the FK values, SIFA achieves a perfect rank of 1 on all metrics, followed by ET as the second best, while BiLSTM-AADC and ANN perform the poorest, as confirmed by Fig. 21, which displays the average FK of each model across all performance metrics. To assess the statistical significance of these improvements, the p-values comparing SIFA with the other models are reported in the same table. Inspection of this table reveals that the proposed model was able to achieve statistically significant improvements. However, these values did not reflect the magnitude of the difference. Therefore, the effect size was estimated and reported in Table 8. This table shows that the effect size of SIFA with all algorithms is large since it exceeds 0.5, indicating that the improvements achieved by SIFA are both substantial and meaningful. Finally, the average computational cost of each model on both the training and testing datasets is computed and reported in Fig. 22. The results show that SIFA has a slightly higher computational cost than the other ML models and is significantly lower than that of the DL model.

**Table 7:** Results of the proposed SIFA and some competing models on DS3.

Metrics		SIFA	ET	PBNN	RF	LGB	LSTM	BiLSTM-AADC	ANN
**MSE**	Best	**5.9857E + 03**	6.0790E + 03	7.3622E + 03	6.7776E + 03	7.0863E + 03	1.1103E + 04	1.6432E + 04	1.6939E + 04
	Avg	**6.2715E + 03**	6.4032E + 03	8.3710E + 03	7.0524E + 03	7.4221E + 03	1.2073E + 04	1.8062E + 04	1.7678E + 04
	Worst	**6.6264E + 03**	6.7197E + 03	9.0821E + 03	7.4502E + 03	7.7603E + 03	1.3411E + 04	2.2434E + 04	1.8518E + 04
	SD	228.45	237.92	555.93	214.63	**212.97**	670.80	1996.07	537.26
	PV		**0.00195**	**0.00195**	**0.00195**	**0.00195**	**0.00195**	**0.00195**	**0.00195**
	FK	**1.00**	2.00	4.80	3.00	4.20	6.00	7.50	7.50
**RMSE**	Best	**7.7368E + 01**	7.7968E + 01	8.5803E + 01	8.2326E + 01	8.4180E + 01	1.0537E + 02	1.2819E + 02	1.3015E + 02
	Avg	**7.9181E + 01**	8.0008E + 01	9.1447E + 01	8.3970E + 01	8.6144E + 01	1.0984E + 02	1.3422E + 02	1.3294E + 02
	Worst	**8.1402E + 01**	8.1974E + 01	9.5300E + 01	8.6314E + 01	8.8093E + 01	1.1581E + 02	1.4978E + 02	1.3608E + 02
	SD	1.44	1.49	3.08	**1.27**	1.24	3.04	7.22	2.02
	PV		**0.00195**	**0.00195**	**0.00195**	**0.00195**	**0.00195**	**0.00195**	**0.00195**
	FK	**1.00**	2.00	4.80	3.00	4.20	6.00	7.50	7.50
**MAE**	Best	**2.3459E + 01**	4.3649E + 01	5.6422E + 01	5.1214E + 01	4.5277E + 01	1.1587E + 02	1.1598E + 02	1.0804E + 02
	Avg	**2.3989E + 01**	4.4788E + 01	5.8233E + 01	5.2651E + 01	4.7259E + 01	1.3610E + 02	1.2659E + 02	1.2137E + 02
	Worst	**2.4670E + 01**	4.6365E + 01	6.0751E + 01	5.5080E + 01	4.9529E + 01	1.5335E + 02	1.3455E + 02	1.3289E + 02
	SD	**0.434**	0.704	1.274	1.406	1.308	10.649	6.359	7.523
	PV		5.0620E-03	5.0620E-03	5.0620E-03	5.0620E-03	5.0620E-03	5.0620E-03	5.0620E-03
	FK	**1.00**	2.00	5.00	4.00	3.00	7.70	6.80	6.50
**R** ^**2**^	Best	**9.3330E-01**	9.3236E-01	9.0945E-01	9.2501E-01	9.2188E-01	8.6501E-01	7.7418E-01	8.1385E-01
	Avg	**9.3712E-01**	9.3580E-01	9.1607E-01	9.2929E-01	9.2558E-01	8.7893E-01	8.1891E-01	8.2275E-01
	Worst	**9.4012E-01**	9.3873E-01	9.2589E-01	9.3163E-01	9.2934E-01	8.8929E-01	8.3493E-01	8.3110E-01
	SD	**2.29E-03**	2.41E-03	5.54E-03	2.09E-03	2.20E-03	7.14E-03	2.00E-02	5.74E-03
	PV		**0.00195**	**0.00195**	**0.00195**	**0.00195**	**0.00195**	**0.00195**	**0.00195**
	FK	**1.00**	2.00	4.80	3.00	4.20	6.00	7.50	7.50

*Bold values in the PV row indicate the significance of the SIFA improvements, while Bold values in the remaining rows stand for the best outcomes. All reported values are computed on the held-out test set.

**Table 8:** Effect size (r), computed from the WSR test using $\:Z/\surd\:N$, for SIFA compared to competing models on DS3.

MSE	ET	PBNN	RF	LGB	LSTM	BiLSTM-AADC	ANN
	0.886405	0.886405	0.886405	0.886405	0.886405	0.886405	0.886405
RMSE	0.273980	0.273980	0.273980	0.273980	0.886405	0.886405	0.886405
MAE	0.886405	0.886405	0.886405	0.886405	0.886405	0.886405	0.886405
R^2^	0.886405	0.886405	0.886405	0.886405	0.886405	0.886405	0.886405

## Conclusion and future work

This study presents SIFA, a new two-stage forecasting approach designed to accurately predict SI, thereby improving the stability and efficiency of PV power plants. In the first stage, the SFS technique determines the best feature subset for accurate SI prediction, with RF assessing different subsets to choose the most effective one. To fine-tune the RF estimators, we propose an improved variant of MRFO (IWMRFO) that employs chaotic maps instead of random generators to better balance exploration and exploitation, helping to avoid local optima and accelerating convergence. The second stage integrates three ML models—HR, ET, and XGB—using a weight vector optimized by IWMRFO, ensuring optimal weights that boost the proposed SIFA approach’s training and generalization performance. This approach is validated on three well-known datasets—San Diego, Islamabad, and the NASA SI—and benchmarked against several other models using metrics such as RMSE, MAE, MAPE, MSE, and R². According to the experimental findings, SIFA significantly outperforms all other models across all evaluation metrics on the three datasets, with improvements from 1% to 95%. These improvements demonstrate the strong potential of the proposed SIFA approach to enhance the efficiency and reliability of PV-based power systems, reduce operational costs, and support the integration of renewable energy sources into the electrical grid. Although SIFA surpasses several competing models, it requires more computational resources than basic ML models, which represents a primary limitation that should be tackled in future research to enhance efficiency. Additionally, because the model combines three base models with different hyperparameters, thorough tuning is computationally demanding, constituting a second limitation of the proposed SIFA that should also be addressed in the future. Additionally, Future work will investigate the performance of the proposed SIFA approach for various prediction problems, including PEMFC remaining useful life estimation, wind energy prediction, CO₂ emission forecasting, and energy consumption forecasting for non-residential buildings.

## Data Availability

The datasets generated during and/or analyzed during the current study are available from the corresponding author upon reasonable request.
